# *Caulobacter crescentus* Hfq structure reveals a conserved mechanism of RNA annealing regulation

**DOI:** 10.1073/pnas.1814428116

**Published:** 2019-05-10

**Authors:** Andrew Santiago-Frangos, Kathrin S. Fröhlich, Jeliazko R. Jeliazkov, Ewelina M. Małecka, Giada Marino, Jeffrey J. Gray, Ben F. Luisi, Sarah A. Woodson, Steven W. Hardwick

**Affiliations:** ^a^Cell, Molecular and Developmental Biology and Biophysics Program, Johns Hopkins University, Baltimore, MD 21218;; ^b^Thomas C. Jenkins Department of Biophysics, Johns Hopkins University, Baltimore, MD 21218;; ^c^Department of Biology I, Microbiology, Ludwig Maximilian University Munich, 82152 Martinsried, Germany;; ^d^Program in Molecular Biophysics, Johns Hopkins University, Baltimore, MD 21218;; ^e^Department of Biology I, Plant Molecular Biology, Ludwig Maximilian University Munich, 82152 Martinsried, Germany;; ^f^Department of Chemical and Biomolecular Engineering, Johns Hopkins University, Baltimore, MD 21218;; ^g^Department of Biochemistry, University of Cambridge, CB2 1GA Cambridge, United Kingdom

**Keywords:** Hfq, *Caulobacter*, sRNA, RNA–protein interaction, natively unstructured protein

## Abstract

We have solved the X-ray crystal structure of the RNA chaperone protein Hfq from the alpha-proteobacterium *Caulobacter crescentus* to 2.15-Å resolution, resolving the conserved core of the protein and the entire C-terminal domain (CTD). The structure reveals that the CTD of neighboring hexamers pack in crystal contacts, and that the acidic residues at the C-terminal tip of the protein interact with positive residues on the rim of Hfq, as has been recently proposed for a mechanism of modulating RNA binding. De novo computational models predict a similar docking of the acidic tip residues against the core of Hfq. We also show that *C. crescentus* Hfq has sRNA binding and RNA annealing activities and is capable of facilitating the annealing of certain *Escherichia coli* sRNA:mRNA pairs in vivo. Finally, we describe how the Hfq CTD and its acidic tip residues provide a mechanism to modulate annealing activity and substrate specificity in various bacteria.

## Significance

In many bacteria, the RNA chaperone protein Hfq binds to hundreds of small noncoding RNAs and improves their efficacy by aiding base pairing to target mRNAs. Hfq proteins contain a variable C-terminal domain (CTD), usually structurally disordered, which was recently demonstrated to inhibit Hfq from mediating nonspecific RNA annealing. We obtained a new structure that shows how this inhibition is achieved in *Caulobacter crescentus* Hfq. The structural data and chaperone assays provide an initial view of the little-known mechanism of small RNA regulation in *Caulobacter*. In addition, this work demonstrates how the Hfq CTD has evolved to meet the needs for species-specific selectivity in RNA binding and pairing of regulatory RNAs with cognate targets.

The RNA chaperone protein Hfq, a member of the Lsm/Sm protein superfamily, is widespread in the bacterial kingdom, where it facilitates gene regulation by small noncoding RNAs (sRNAs) and other types of posttranscriptional control (1, 2). The role of Hfq in sRNA regulation has been well studied in gamma-proteobacteria, such as *Escherichia coli* and *Salmonella* spp.; however, little is known of its function in the distantly related alpha-proteobacteria. Deletion of *hfq* from the genome of the freshwater-dwelling alpha-proteobacterium *Caulobacter crescentus* results in a severe loss of fitness and an elongated cell morphology, most likely due to limited synthesis of peptidoglycan precursors (3). The precise role of the *C. crescentus* Hfq (*Cc* Hfq hereinafter) protein as an RNA chaperone in this process is unclear, although in RNA sequencing experiments, the levels of hundreds of transcripts, including several mRNAs encoding metabolic genes, were altered in the absence of *hfq* (3). How the level of Hfq protein in *C. crescentus* is regulated is also poorly understood, although the evidence suggests that expression of the *hfq* gene is partially controlled by the response regulator SpdR (4). To date, there are no reported direct sRNA or mRNA targets of *Cc* Hfq, and its RNA annealing activity is undetermined.

The structure of the Hfq “core” region from several different bacterial species has now been solved, and in all cases a conserved hexameric ring structure has been observed. Most RNA binding and annealing data are derived from observations with *Escherichia coli* Hfq (*Ec* Hfq hereinafter) and closely related homologs (5). These studies have identified the distal and proximal faces and a basic patch on the circumferential rim as surfaces that engage RNA. A pore on the proximal face prefers uridine stretches, and the distal face recognizes A-R-N repeats, where R is a purine and N is any nucleotide (6). Sequence and structural analyses of bacterial and archaeal Hfq proteins suggest that polyuridine recognition by the proximal pore is strongly conserved (7–10) and likely to be maintained in most Hfq homologs, whereas the more variable distal face may have different RNA-binding preferences in different species (8, 11–13). The basic patch on the rim of Hfq is necessary to facilitate the annealing of sRNAs to target transcripts in vitro (14, 15). Annealing activity corresponds with the number of arginines in the basic patch (16), suggesting that Hfq proteins lacking these arginine residues may have little activity for pairing partner RNAs, but instead could participate in other aspects of RNA metabolism (17–20).

Appended to the core of Hfq is a short variable N-terminal extension (NTE) that is usually only four amino acids long (5) and a longer variable C-terminal domain (CTD) that is predicted to be unstructured (21). The structure of the Hfq CTD has not been fully resolved in previous Hfq crystal structures and in many cases has been removed from the protein constructs before crystallization (22, 23). Solution structural data for *E. coli* and *Vibrio cholerae* Hfq also indicate partial disorder in the CTDs (24, 25).

**Fig. 1.**
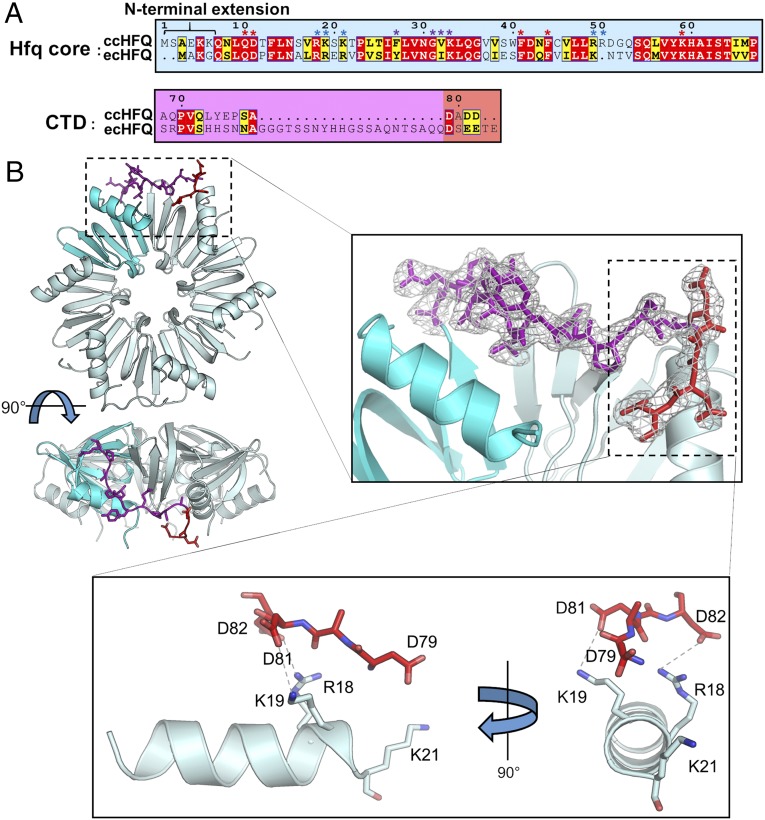
The structure of *Cc* Hfq. (*A*) Sequence alignment of *Cc* Hfq and *Ec* Hfq. Residues forming the Hfq core have a cyan background, residues contained in the C-terminal domains have a purple background, and acidic tip residues have a salmon background. *E. coli* residues implicated in RNA binding to proximal face, distal face, and rim of Hfq are marked with red, purple, and blue asterisks, respectively. Alignment was generated by ENDscript (66). (*B*) The crystal structure of *Cc* Hfq. (*Upper Left*) A hexamer of Hfq shown in two views as a cartoon, with protomers in cyan and a single protomer in a darker shade of cyan. The CTD is shown as purple sticks, and DADD residues at the C terminus are shown as red sticks. (*Upper Right*) Zoomed-in view of the CTD, with the 2Fo-Fc experimental electron density map shown at 1σ level. (*Lower*) Zoomed-in view of the interaction between the acidic tip residues (red sticks) and the positive rim residues (cyan sticks). Electrostatic interactions are shown as dashed lines.

Given the lack of detailed structural information available for the CTD, the roles of these Hfq appendages are less well understood. The CTD of Hfq in *E. coli* has recently been proposed to autoinhibit RNA annealing activity, as acidic residues at the very C-terminal tip of the protein mimic the nucleic acid phosphodiester backbone to displace RNA from the rim (26, 27). Like other disordered protein regions, the CTD of Hfq diverges at a faster rate than the structured Sm-like core, via nonconservative substitutions and indels (28–30). The *Cc* Hfq CTD is less than one-half the length of the *Ec* Hfq CTD (15 aa vs. 38 aa) yet retains an acidic motif at the CTD tip (-DADD), similar to that seen in *Ec* Hfq (-DSEETE) (Fig. 1*A*).

We have solved the X-ray crystal structure of *Cc* Hfq to 2.15-Å resolution, including the structure of the entire length of the Hfq CTD. The structure reveals how the acidic tip residues pack against the positive core residues, in agreement with predictions (27), and shows the CTD packing against the CTD of neighboring hexamers in an antiparallel arrangement. We have used this crystal structure to validate Rosetta FloppyTail (31), a de novo modeling algorithm for disordered regions of proteins. Finally, we evaluate the RNA binding and annealing activity of *Cc* Hfq and show structurally and biochemically that the CTD of Hfq provides a mechanism to regulate annealing activity and substrate specificity across bacterial species.

## Results

### Crystal Structure of *Cc* Hfq.

Purified full-length *Cc* Hfq was crystallized, and the structure was solved using the *Ec* Hfq core hexamer as a molecular replacement search model (22). Four Hfq hexamers could be modeled in the crystal asymmetric unit, packing in an unusual arrangement with the hexamers positioned orthogonal to one another to generate an “open square” (*SI Appendix*, Fig. S1*A*). From the electron density maps, residues in the short CTD of *Cc* Hfq could be visualized for several protomers within the asymmetric unit, in some cases allowing for modeling of the entire length of the CTD with a high degree of confidence (Fig. 1*B*). On modeling the *Cc* Hfq CTD, it became apparent that these extensions pack against the CTD of neighboring hexamers in an antiparallel manner (*SI Appendix*, Fig. S1*B*). The *Cc* Hfq CTDs that are not directly involved in contacting neighboring hexamers are partially disordered.

From the structure of *Cc* Hfq, it is clear that the acidic tip residues (DADD; residues 78–82) pack against the positive rim residues of the proximal face (R18, K19, and K21), with few direct contacts to the arginine patch on the distal side of the rim (R49 and R50) (Fig. 1*B*). Specifically, aspartates 81 and 82 are positioned to make electrostatic interactions with lysine 19 and arginine 18, respectively. Interestingly, the side chain of aspartate 79 is facing away from the positive rim residues on the neighboring protomer, but instead is within hydrogen-bonding distance of arginine 49 on a neighboring hexamer (*SI Appendix*, Fig. S1*B*). It should also be noted that lysine 21 from the positive rim patch does not directly interact with the acidic tip residues in the crystal structure, but instead makes a hydrogen-bonding interaction with arginine 49 within the same protomer (*SI Appendix*, Fig. S1*C*). Finally, glutamate 75 from within the CTD forms a hydrogen bond with arginine 49 and serine 39 on the neighboring protomer within the Hfq hexamer (*SI Appendix*, Fig. S1*C*). These interactions agree with the proposed autoinhibitory mechanism in which acidic tip residues pack against conserved positive rim residues on the core of Hfq to compete with nucleic acid binding to the rim and disfavor spurious RNA annealing events (27).

The well-ordered CTD–rim interactions observed in our structure suggest that the short *Cc* Hfq CTD may provide even stronger autoinhibition than the longer and more flexible *Ec* Hfq CTD.

### De Novo Modeling of the *Cc* Hfq Tail.

To better understand whether the CTD–core interactions captured in our crystals could occur in solution, we modeled the flexible NTEs and CTDs of *Cc* Hfq using Rosetta FloppyTail, a de novo computational modeling algorithm (31) that has been previously used to predict interaction energies in the *Ec* Hfq CTD with good correlations to in vitro mutagenesis assays (27). To ensure that the results were not biased, the calculations were performed without prior knowledge of the *Cc* Hfq crystal structure.

**Fig. 2.**
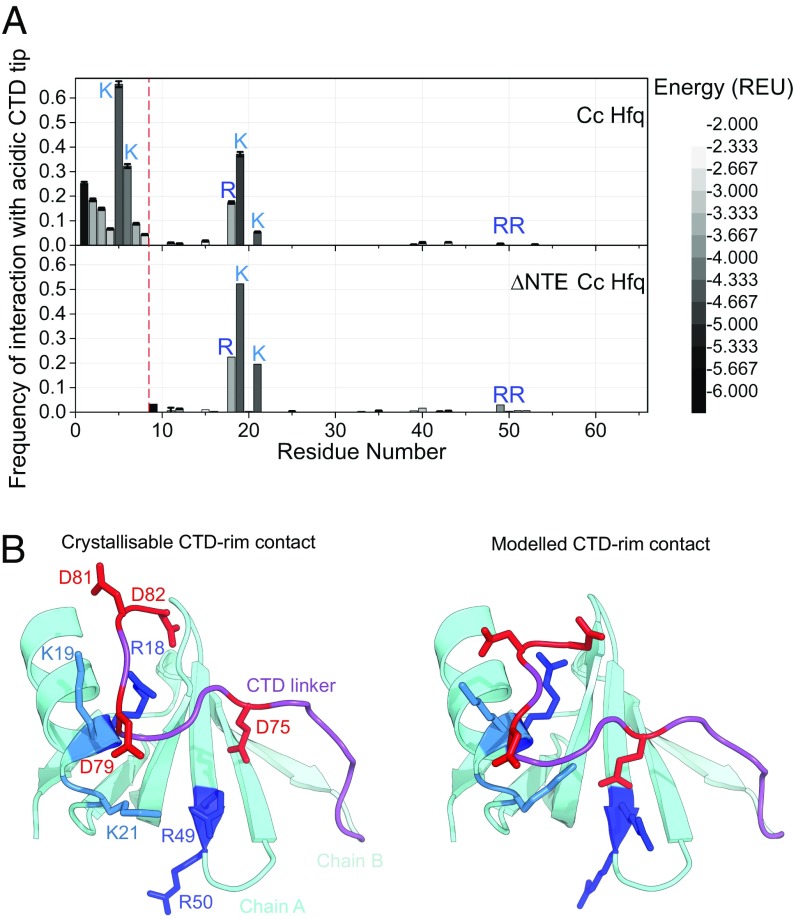
Computational modeling of *Cc* Hfq CTD confirms interactions observed in crystallo. (*A*) Observed frequency of favorable (E <−2.0 REU) residue-to-acidic-CTD interactions in low-energy FloppyTail models. (*Upper*) Full-length *Cc* Hfq. (*Lower*) *Cc* Hfq with residues of the NTE deleted (ΔNTE). On exclusion of the NTE in silico, only the basic core residues retain interactions with the CTD. Error bars show ±1 SD, as computed by bootstrap resampling. (*B*) Side-by-side comparison of crystallized and modeled CTD-core contacts. The acidic tip residues in the CTD of the adjacent monomer (chain B, light cyan) contact the basic rim residues of the monomer in the forefront (chain A, cyan). For clarity, the NTE and CTD of chain A and most of chain B except the last β-sheet and CTD are omitted. CTDs are shown in purple; lysines, in sky blue; arginines, in royal blue; and CTD acidic residues, in red.

The overall basicity of the rim is conserved between *Cc* and *Ec* Hfqs (Fig. 1*A*); however, the arginines are distributed more toward the rim-distal face in *Cc* Hfq (*SI Appendix*, Fig. S2). Among the lowest-energy fraction of models generated in our simulations (1% of all models, sorted by energy), acidic residues at the CTD of *Cc* Hfq were found to frequently form energetically favorable contacts with basic residues arginine 18, lysine 19, and lysine 21 on the proximal-rim interface (Fig. 2*A*), even when the basic NTEs were excluded from the model. In contrast, few contacts were observed to arginines 49 and 50, which are solvent-exposed but lie toward the distal side of the rim. Nearly all of the lowest-energy fraction of models had at least one CTD in contact with the rim, and many of the modeled CTD conformations closely resembled (∼2 Å Cα rmsd) the crystallized CTD–rim interaction (Fig. 2*B* and *SI Appendix*, Figs. S3 and S4). In silico mutation of both arginine 18 and lysine 19 to alanine ablated interactions of acidic CTD residues with these positions on the core (*SI Appendix*, Fig. S5). These results illustrate the predictive power of Rosetta FloppyTail and suggest that the crystallized CTD–rim interaction is likely to occur in solution rather than being an artifact of crystal lattice packing.

### *Ec* Hfq Binds sRNAs More Avidly than *Cc* Hfq.

The sequence-specific RNA binding sites of the Sm-like core of *Cc* Hfq bear strong sequence conservation to *Ec* Hfq (Fig. 1*A*) and are expected to bind U-rich RNA at the proximal face and A-rich RNA at the distal face. Docking of poly(A) RNA bound to *Ec* Hfq (6) onto the structure of *Cc* Hfq shows that the RNA can be accommodated on the distal face of the protein. Using fluorescence anisotropy, we observed that the *Cc* Hfq protein can indeed bind to A18-FAM RNA with high affinity (*K*_d_ ≤2 nM Hfq_6_; *SI Appendix*, Fig. S6), comparable to that of *Ec* Hfq.

**Fig. 3.**
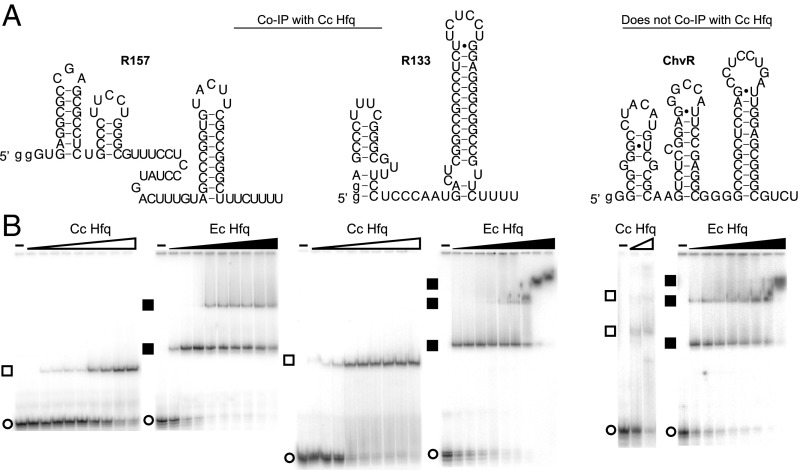
sRNA binding experiments. (*A*) *C. crescentus* sRNAs used in this study. R157 and R133 coimmunoprecipitate with *Cc* Hfq, whereas ChvR is a *C. crescentus* sRNA that does not coimmunoprecipitate with *Cc* Hfq and regulates its targets independently of Hfq (36). (*B*) Native gel EMSAs for *Cc* and *Ec* Hfqs. Free sRNA bands are indicated with an open circle, and Hfq complexes are indicated with open (*Cc*) and solid (*Ec*) squares. *SI Appendix*, Fig. S8 provides images of assays with other Hfq variants, and *SI Appendix*, Table S6 lists the protein concentrations used in the EMSAs.

The distantly related *E. coli* and *C. crescentus* genomes possess very different GC content, of 51% (32) and 67% (33) respectively. Therefore, the *C. crescentus* genome is expected to encode sRNAs with more stable secondary structures, which may impact Hfq–sRNA interactions. We first compared the binding of *Cc* Hfq and *Ec* Hfq proteins to three sRNAs from *C. crescentus*. The genome of *C. crescentus* encodes four homologs (CCNA_R0014, CCNA_R0133, CCNA_R0143, and CCNA_R0157) of the alpha-proteobacterial αr15 family of noncoding RNAs, which also includes the Hfq-binding sRNAs AbcR1/2 in *Agrobacterium tumefaciens* and *Sinorhizobium meliloti*, respectively (34, 35). Likewise, CCNA_R0014 has recently been recovered in coimmunoprecipitation experiments with 3× FLAG-tagged *Cc* Hfq (36), and the paralog sRNAs CCNA_R0157 (R157) and CCNA_R0133 (R133) were expected to bind Hfq with high affinity. These GC-rich (60–72%) *C. crescentus* sRNAs are predicted to form highly stable stem-loops (Fig. 3*A*) and possess sequence and structural characteristics of *E. coli* class I sRNAs that predominantly engage the rim and proximal face via a U/A-rich region upstream of the terminator stem-loop and a single-stranded U-rich 3′ end (14, 37–40). A third sRNA, CCNA_R0100 (ChvR), was not coprecipitated with 3× FLAG-tagged *Cc* Hfq (36), and so was included as a negative control. ChvR is predicted to form a stable secondary structure but lacks U/A- and U-rich sequence motifs.

Native gel electrophoretic mobility shift assays (EMSAs) showed that *Cc* Hfq formed stable complexes with R157 and R133 sRNAs (Fig. 3*B* and *SI Appendix*, Table S1). However, *Cc* Hfq had very weak affinity for ChvR, as expected (Fig. 3*B*). In contrast, *Ec* Hfq bound all three *C. crescentus* sRNAs more tightly than *Cc* Hfq and exhibited little preference for R157 and R133 compared with the “nonspecific” sRNA ChvR (Fig. 3*B* and *SI Appendix*, Table S1). Thus, *Cc* Hfq binds *C. crescentu*s sRNAs less avidly than *Ec* Hfq, and with greater discrimination. We noted that *Ec* Hfq also has a greater propensity than *Cc* Hfq to form higher-order complexes with sRNAs, which are thought to contain a second Hfq hexamer (Fig. 3*B*). This observation is addressed in *Discussion*.

**Fig. 4.**
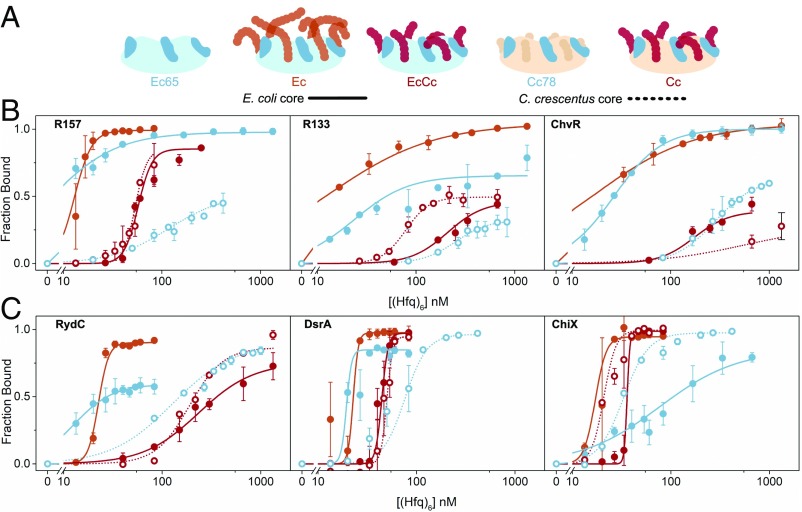
Equilibrium interactions of Hfq and sRNAs. (*A*) Hfq variants used in the assays. *Ec* Hfqs (solid lines; filled circles) or *Cc* Hfqs (dotted lines; open circles) bearing no acidic CTD tip (light blue), a CTD with a distantly tethered acidic tip residues (orange), or a CTD with a closely tethered acidic tip residues (red). All Hfq variants used in for RNA binding and RNA annealing experiments lack exogenous affinity tags. (*B* and *C*) Binding curves for *Ec* Hfq (solid lines) or *Cc* Hfq (dotted lines) variants with *C. crescentus* and *E. coli* sRNAs, respectively. Symbols for single representative trials are shown. The fraction bound was fit to single binding isotherms, normalized for the maximum fraction bound at saturating Hfq. *K*_d_ values and Hill coefficients for each fit are listed in *SI Appendix*, Table S1.

To further explore how *Ec* Hfq and *Cc* Hfq proteins recognize their endogenous sRNAs, we examined the binding of *Ec* and *Cc* Hfq variants to the three *C. crescentus* sRNAs above and to four *E. coli* sRNAs: RydC, DsrA, RybB, and ChiX (*SI Appendix*, Fig. S7). *Ec* Hfq had a greater affinity than *Cc* Hfq for all seven sRNAs tested (Fig. 4 and *SI Appendix*, Fig. S8 and Table S1). However, each sRNA interacted slightly differently with the *Ec* and *Cc* Hfq proteins. The *E. coli* sRNAs RydC, DsrA, and RybB are class I sRNAs that form sequence-specific contacts with the proximal face and rim of Hfq (39). *Cc* Hfq formed particularly weak complexes with RydC sRNA (*K*_d_ = 206 nM; red open symbols in Fig. 4*C*), an sRNA that must partially unfold to interact with the Hfq rim (41). The poor binding of *Cc* Hfq to RydC may be explained structurally by a clash of the RNA with the acidic CTD tip (*SI Appendix*, Fig. S9). In contrast, *Cc* Hfq and *Ec* Hfq bound ChiX sRNA with similar affinity. ChiX is a class II sRNA that contains an ARN motif and makes sequence-specific contacts with both the proximal and distal faces of Hfq (38–40), and thus is likely to be less susceptible to competition for the rim by the CTDs.

### *Cc* Hfq CTD Increases the Selectivity of sRNA Binding.

We next asked how the CTD contributes to these differences by examining sRNA binding of *Ec* Hfq core (*Ec*65; residues 1–65 only), *Cc* Hfq lacking the acidic tip residues (*Cc*78; residues 1–78 only), and a chimera of *Ec* Hfq core and *C. crescentus* Hfq CTD (*EcCc* Hfq; residues 1–67 of *Ec* Hfq fused to residues 71–82 of *Cc* Hfq) (Fig. 4*A*). We found that truncation of either the entire CTD (*Ec*65) or the acidic CTD tip (*Cc*78) decreased the apparent affinities and cooperativity of Hfq binding to all the sRNAs with single-stranded Hfq binding sites (R157, DsrA, RybB, and ChiX). Conversely, removal of the CTD increased the binding of Hfq to RydC and to the non–Hfq-specific ChvR sRNA (blue symbols in Fig. 4*B* and *SI Appendix*, Table S1).

When the autoinhibitory *Cc* Hfq CTD was appended to the *Ec* Hfq core (compare *EcCc* Hfq to *Ec65* and *Ec* Hfqs), the average affinity for sRNAs was reduced and comparable to that of *C. crescentus* proteins (compare *EcCc* Hfq to *Cc* Hfq). The *Cc* Hfq CTD also restored the apparent cooperativity of Hfq binding, as shown by the transition slopes in Fig. 4 *B* and *C*. Thus, the *Cc* Hfq CTD appears to inhibit sRNA binding more strongly than the *Ec* Hfq CTD. This increased autoinhibition by the shorter *Cc* Hfq CTD agrees with our previous observation that a shortened CTD increases autoinhibition due to a higher local concentration of the acidic CTD tip around the rim of Hfq (27).

It was initially surprising that removal of the C-terminal residues in *Cc*78 made binding of Hfq-specific R157 and R133 sRNAs less favorable, while making binding of non–Hfq-specific ChvR more favorable. Further inspection of the EMSA results showed that *Cc*78 Hfq complexes were more heterogeneous than *Cc* Hfq complexes (*SI Appendix*, Fig. S8). For example, the band for the ChvR-*Cc*78 Hfq complex was diffuse rather than sharp, and RydC and DsrA formed a triplet of complexes with differing mobilities, perhaps reflecting alternative RNA or protein conformations. In addition, we observed diffuse ^32^P below the main Hfq·sRNA band that presumably arose from dissociation of Hfq during electrophoresis. The quantity of ^32^P-labeled RNA from these dissociated complexes was measurably higher for binding reactions with *Ec*65 and *Cc*78 lacking the acidic CTD than for binding reactions with full-length *Ec*, *EcCc*, and *Cc* Hfq proteins (*SI Appendix*, Fig. S10). As discussed below, these observations suggest that the Hfq CTD favors specific modes of sRNA binding at the expense of nonspecific sRNA–Hfq interactions, improving the kinetic stability of the remaining Hfq-sRNA complexes and increasing the selectivity for sRNAs with Hfq recognition motifs, such as R157 or ChiX. The selection of kinetically stable Hfq-sRNA complexes increases the proportion of Hfq-sRNA complexes that are represented in the EMSA data.

### CTD of *Cc* Hfq Is a Potent Autoinhibitor of RNA Annealing.

**Fig. 5.**
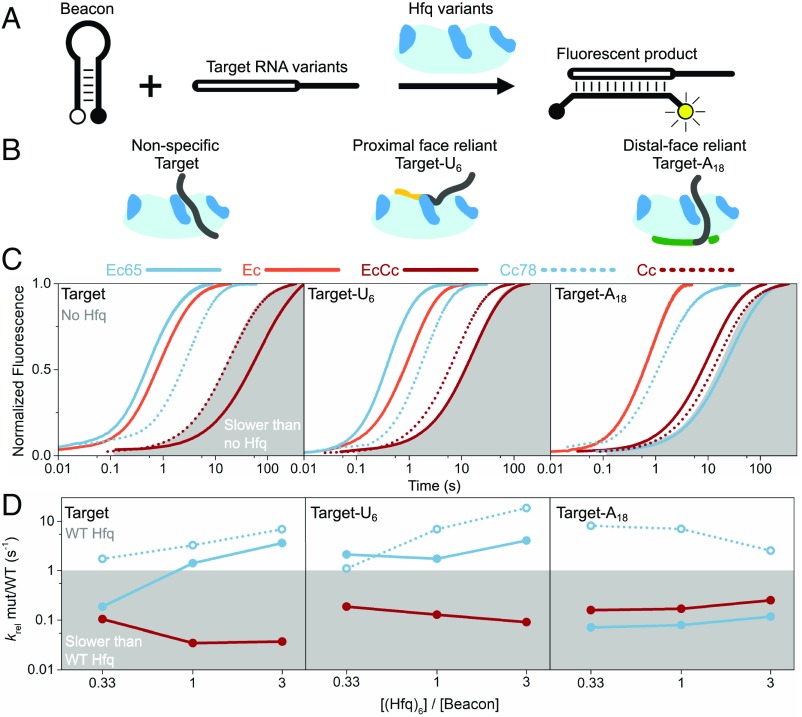
In vitro RNA annealing activity of *Cc* Hfq. (*A*) Molecular beacon assay for annealing of 16 nt synthetic RNA (Target) that binds Hfq nonspecifically (1 µM). (*B*) Target RNA was extended with U_6_ or A_18_ (Target-U6 or Target-A18) that specifically bind the proximal or distal face of Hfq, respectively. (*C*) Stopped-flow fluorescence progress curves for annealing reactions of Target (*Left*), Target-A18 (*Center*), or Target-U6 (*Right*) in 50 nM Hfq hexamer. *Ec* Hfq (solid lines) and *Cc* Hfq (dotted lines) variants are indicated in the key; the basal, no Hfq reaction is indicated by gray shading. Progress curves were normalized to the maximum fluorescence change after a maximum of 500 s. Raw data were down-sampled to enable plotting *C. crescentus* datasets as dotted lines. An exception is that Ec65 sequesters Target-A18 in an inactive complex in this assay (*SI Appendix*, Fig. S11), as explained in *SI Appendix*. (*D*) Effect of the CTD on annealing. Observed annealing rate constants (*k*_obs_) in the presence of EcCc, Ec65, and Cc78 relative to *k*_obs_ for the parental WT protein (*Ec* or *Cc*; gray shading). Kinetics were measured in 16.7, 50, or 150 nM Hfq hexamer for three targets as in *B*. Rate constants are the average of at least five replicates; propagated error bars are smaller than the symbols plotted.

The CTD of *Ec* Hfq limits its annealing of minimal RNAs, which we propose is due to competition between the CTD and RNA binding to the basic patches on the rim of the protein (26). According to our model (27), the shorter CTD of *Cc* Hfq should inhibit annealing more strongly than the longer *Ec* Hfq CTD. To evaluate the contributions of the CTD to RNA annealing, we carried out RNA annealing assays with the same *Cc* and *Ec* Hfq variants used for the sRNA binding experiments (Fig. 5 and *SI Appendix*, Fig. S13). The ability of Hfq to accelerate base pairing between an RNA molecular beacon and three different complementary RNAs (Fig. 5*A*) was measured by stopped-flow fluorescence spectroscopy (42). These minimal RNAs, which lack secondary structure but retain Hfq binding sequences, have been used to compare the basal annealing activities of other bacterial Hfq proteins (16). The shortest (16 nt) target RNA binds the rim of Hfq weakly and nonspecifically (43). Target-U6 RNA binds tightly and specifically to the proximal face of Hfq, whereas Target-A18 RNA specifically binds to the distal face of Hfq (Fig. 5*B*).

*Cc* Hfq was far less active than *Ec* Hfq on all the minimal RNAs tested (Fig. 5*C*). Whereas *Ec* Hfq accelerated annealing by approximately 10-fold, *Cc* Hfq barely increased the annealing rates of Target-U6 and Target-A18 when *Cc* Hfq hexamer and RNA beacon were present in equimolar amounts (Fig. 4*B*; orange and red vs. gray). The low activity of *Cc* Hfq was not due to poor RNA binding, as *Cc* Hfq can recognize single-stranded U_6_ and A_18_ tails (Fig. 3 and *SI Appendix*, Fig. S6).

We next considered whether the annealing activity of *Cc* Hfq was inhibited by its CTD. We visualized the effect of the CTD on the chaperone activity of Hfq by comparing the annealing rates of the CTD variants with their respective wild-type parents at three different Hfq concentrations (Fig. 5*D* and *SI Appendix*, Fig. S13). Removal of the acidic CTD residues from either protein (*Ec*65 or *Cc*78) increased the annealing activity by 2- to 10-fold on these minimal RNAs (Fig. 5*D*; solid blue and dotted blue lines). *Cc*78 generally exhibited a greater relief of autoinhibition than *Ec*65. Although the *Ec*65 core was more active than *Cc*78 for annealing Target and Target-U6, the chimeric *EcCc* Hfq was less active than the wild-type *Ec* Hfq and produced less dsRNA product (red, Fig. 5*D*). An exception to this trend is that *Ec*65 forms inactive complexes when premixed with a twofold molar excess of an A-rich target (*SI Appendix*, Fig. S11). Thus, *Cc* Hfq is capable of annealing minimal RNAs, but this activity is inhibited by its C-terminal acidic residues. In addition, this comparison suggests that the *Cc* Hfq CTD inhibits nonspecific RNA annealing more strongly than *Ec* Hfq CTD (27).

### *Cc* Hfq Can Facilitate sRNA-mRNA Regulation in Vivo.

**Fig. 6.**
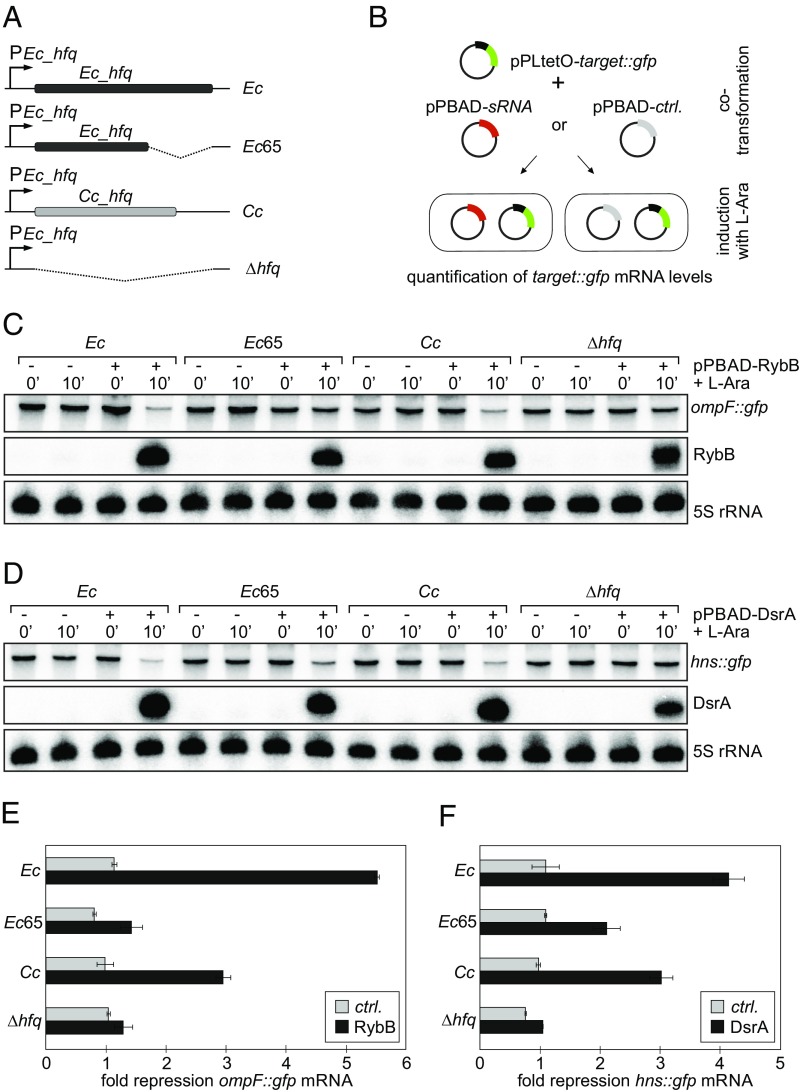
In vivo RNA annealing assays. (*A*) Schematic of *E. coli* mutant strains used in RNA annealing assays. All Hfq variants are expressed from the MC4100 *hfq* locus under control of the native *Ec_hfq* promoter. Expression of the Hfq variants was confirmed by Northern blot analysis and LC-MS/MS (*SI Appendix*, Fig. S14) (*B*) Reporter assay to monitor posttranscriptional activity of sRNAs. *E. coli* strains were cotransformed with a translational fusion of sRNA target transcripts to *gfp* (*target::gfp*; under control of the constitutive P_LtetO1_ promoter) and either an empty control vector or a plasmid expressing an sRNA from the arabinose-inducible P_BAD_ promoter. RNA collected before and at 10 min postinduction with l-arabinose (final concentration 0.01%) was analyzed on Northern blots, and *target::gfp* mRNA levels were quantified. (*C* and *D*) Northern blot analysis of *ompF::gfp* mRNA (*C*) and *hns::gfp* mRNA levels (*D*) in strains carrying a control plasmid or in response to pulse induction of RybB (*C*) or DsrA (*D*) sRNAs. Expression of sRNAs was validated using gene-specific probes; 5S rRNA served as a loading control. (*E* and *F*) Quantification of *ompF::gfp* (*E*) or *hns::gfp* mRNA (*F*) repression (at the 10-min time point) from Northern blot analysis as shown in *C* and *D*. Error bars represent the deviation of three biological replicates.

As there are currently no sRNA-mRNA regulatory pathways in *C. crescentus* known to rely on Hfq, we examined whether *Cc* Hfq could be transferred into *E. coli* to facilitate interactions between sRNA-mRNA pairs in vivo. As examples, we investigated down-regulation of *ompF* mRNA by RybB sRNA, down-regulation of *hns* mRNA by DsrA sRNA, and activation of *cfa* mRNA by the sRNA RydC. Using λRed recombination (44), we truncated the coding sequence of *Ec hfq* (*Ec*65) or replaced it completely with the corresponding *C. crescentus* sequence (referred to as *Cc* Hfq) on the chromosome of *E. coli* MC4100 under control of the native *Ec hfq* promoter (Fig. 6*A*).

We then compared sRNA-mediated regulation of the cognate mRNA targets in the presence of *Ec*, *Ec*65, or *Cc* Hfq to an isogenic *hfq* deletion mutant (Fig. 6*B*). To this end, we transformed strains expressing the different Hfq variants with translational fusions of sRNA target transcripts to *gfp* (45). In these reporters (i.e., *ompF::gfp*, *hns::gfp*, and *cfa::gfp*), the 5′ UTR and the first codons of the mRNA targets are fused to the second codon of *gfp*, and expression is driven from the constitutive P_LtetO_ promoter to solely record posttranscriptional regulation. We next introduced either a control vector or a construct to express RybB, DsrA, or RydC from the arabinose-inducible P_BAD_ promoter. We grew all strains to midexponential phase (OD_600_ of 1), collected total RNA samples before and at 15 min after the addition of arabinose, and monitored sRNA induction and *target::gfp* mRNA expression by Northern blot analysis.

Three Hfq-dependent sRNAs—RybB, MicA, and MicL—function as the repressory arms of the RpoE-controlled response to perturbations of outer membrane homeostasis (46–48). Collectively, these sRNAs repress >30 mRNAs in *E. coli* by direct base-pairing, including many transcripts encoding outer membrane porins (OMPs). We monitored down-regulation of the well-characterized target *ompF::gfp* in response to RybB induction, and observed that both *Ec* and *Cc* Hfq mediated repression of the reporter (Fig. 6 *C* and *E*). In contrast, *ompF::gfp* mRNA abundance barely changed in cells expressing truncated *Ec*65 Hfq or the *hfq* null mutant, even though RybB was induced to comparable levels in all strains.

We next analyzed the regulatory capacity of DsrA in our different Hfq variant strains. Induced under low-temperature growth conditions, DsrA represses translation of *hns* mRNA in an Hfq-dependent manner (49–51). Our reporter assay confirmed the requirement of Hfq, as we observed down-regulation of *hns::gfp* mRNA in the presence of *Ec* Hfq but not in the *hfq* null strain (Fig. 6 *D* and *F*). Both truncated *Ec*65 Hfq and *Cc* Hfq were able to mediate DsrA activity, although *hns::gfp* repression was slightly reduced compared with *Ec* Hfq (twofold and threefold repression vs. fourfold repression).

**Fig. 7.**
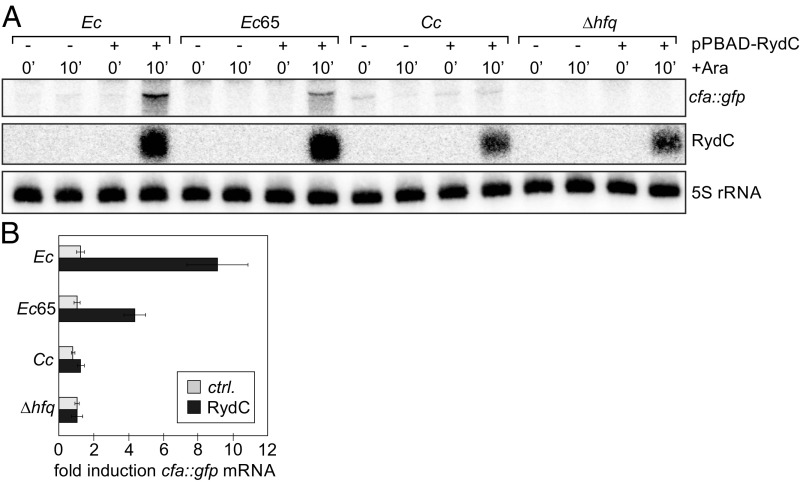
Activation of *cfa::gfp* by RydC depends on *Ec* Hfq. (*A*) Northern blot analysis of *cfa::gfp* mRNA levels in strains carrying a control plasmid or in response to pulse induction of RydC. Expression of RydC was validated using a gene-specific probe; 5S rRNA served as a loading control. (*B*) Quantification of *cfa::gfp* mRNA induction (at the 10-min time point) from Northern blot analysis as shown in *A*. Error bars represent the deviation of three biological replicates.

We also examined the ability of *Cc* Hfq to complement annealing of RydC and its target *cfa* mRNA in vivo. RydC activates expression of the *cfa* transcript in an Hfq-dependent manner via formation of a base-pairing interaction of its conserved 5′ end to an upstream site within the *cfa* 5′ UTR to interfere with mRNA decay (52). Our in vitro binding assays suggested that *Cc* Hfq binds to RydC very poorly compared with *Ec* Hfq (Fig. 4*C*), and in accordance with these results, RydC was not able to activate *cfa::gfp* expression in our reporter assay in the absence of *Ec* Hfq, even though its expression was only mildly affected in the mutant strains (Fig. 7*A*). We also observed that activation of *cfa::gfp* expression by RydC was less effective in the presence of the truncated *Ec*65, suggesting an important contribution of the Hfq CTD in mediating this regulation. Taken together, these results indicate that *Cc* Hfq is able to facilitate the annealing of certain sRNA/mRNA pairs in *E. coli*, such as RybB/*ompF* mRNA and DsrA/*hns* mRNA, which have an accessible Hfq-binding site, but not RydC, which must partly unfold to interact with Hfq. Our crystal structure and in vitro binding results suggest that this difference arises from increased interactions between the *Cc* Hfq CTD and basic residues on the rim of the Hfq hexamer.

## Discussion

The crystal structure of *Cc* Hfq has revealed detailed structural information for the divergent CTDs of bacterial Hfq proteins. Previous sequence analysis, computational modeling, and biochemical assays have suggested that many bacterial Hfq CTDs contain a flexible “linker” of variable length followed by a highly acidic motif that mimics nucleic acid (27). *Cc* Hfq contains a CTD with a naturally short linker but still contains a nucleic acid mimic motif at the C-terminal end (Fig. 1*A*). In the crystal, the majority of the linker region within the CTD is involved in a lattice packing interaction with the linker region of neighboring Hfq hexamers (*SI Appendix*, Fig. S1*B*). Although this may be a favored interaction within the context of the crystal, the self-association of the CTD from *Ec* Hfq has previously been reported in solution (53). Further evidence for the conformation of the CTD seen in the *Cc* Hfq crystal structure being more than a crystal packing artifact came from our de novo blind computational modeling, which showed good agreement in the position of the CTD in relation to the core of Hfq. In this example, the FloppyTail Rosetta modeling algorithm was tested against a crystal structure in a blind test.

In this study, we also assessed the RNA binding and annealing activity of *Cc* Hfq, and these experiments in themselves significantly advance our understanding of the biological role of Hfq across the bacterial kingdom. Our comparative binding results with *Cc* Hfq and *Ec* Hfq toward endogenous and nonendogenous RNA substrates show that *Cc* Hfq recognizes sRNAs comparably to *Ec* Hfq, but with a slightly lower affinity for all RNAs tested. (Binding affinities are summarized in *SI Appendix*, Table S1.) One might assume that Hfq proteins would have evolved to bind with higher affinity to their endogenous substrates, but this does not seem to be the case, as *Ec* Hfq binds with higher affinity than *Cc* Hfq to the specific *C. crescentus* sRNAs tested here. This difference in sRNA affinity is due in part to differences in the Hfq core, because *Ec*65 Hfq binds all sRNAs tested more tightly than *Cc*78 Hfq except for ChiX, which also interacts with the distal face (compare *Ec*65 and *Cc*78 in Fig. 4). Our results also suggest that the CTD is important for ensuring the specificity of *C. crescentus* sRNA-Hfq complexes, as *Cc*78 lacking the C-terminal acidic residues was much more likely to form short-lived complexes that dissociate during gel electrophoresis and complexes with different mobilities. By displacing weakly bound sRNAs, the CTDs not only make binding more selective, but also may reduce the number of allowed binding configurations so that the remaining complexes are conformationally homogeneous. Although the seven sRNAs tested in our study represent only a small fraction of RNAs that interact with Hfq in either species, the results indicate that differences in the Hfq cores and their CTDs both influence the recognition of individual sRNAs. It is also apparent that each Hfq variant used in this study binds RNA ligands somewhat differently, and the determinants for sRNA binding are likely to be complex and remain to be fully understood.

*Ec* Hfq is known to form complexes in which two hexamers bind one RNA, which can tether the two hexamers (41, 54, 55). Interestingly, *Cc* Hfq forms only a single complex on the native gels with all RNAs tested. We envisage that the longer CTD of *Ec* Hfq allows for a “sandwich” complex of Hfq-RNA-Hfq, whereas the limited length of the *Cc* Hfq CTD would prohibit formation of such a complex. It has been previously suggested that these supershifted species are less active than 1:1 Hfq:RNA complexes (56, 57); therefore, Hfq sequences that are less prone to forming these large complexes may retain a higher active fraction of proteins at high Hfq concentrations.

As with previous experiments to shorten the linker of *Ec* Hfq (27), we show that the naturally short linker of *Cc* Hfq imposes a greater degree of autoinhibition of RNA binding and annealing compared with the CTD of *Ec* Hfq, presumably by increasing the local concentration of the acidic motif around the arginine-rich active sites of the Hfq core. The increased specificity imparted by the *Cc* Hfq CTD comes at the cost of decreased RNA annealing activity, at least toward minimal unstructured RNAs (Fig. 5*B* and *SI Appendix*, Fig. S13). To what degree natural *C. crescentus* sRNA and mRNA targets overcome the stronger autoinhibition imposed by the *Cc* Hfq CTD remains to be learned.

Our successful construction of a chimeric Hfq protein composed of the core of *Ec* Hfq and the CTD of *Cc* Hfq showed that the CTD performs the same function of competing for nonspecific RNA interactions in the context of Hfq from the α-proteobacterium *C. crescentus* or the γ-proteobacterium *E. coli*. This modular property of the *Cc* Hfq CTD is due in part to the nature of the CTD–rim interaction, which is rather nonspecific and dominated by electrostatics, reducing the stringency of the CTD–rim interaction in the face of sequence divergence. We believe this observation is of particular importance as it suggests that Hfq CTDs may rapidly diverge to accommodate the acquisition of new sRNAs that could change the organization of regulatory networks.

It is important to also note that the trade-off between specificity and complementation between Hfq proteins from different species correlates with our in vivo annealing data. In the example of RybB sRNA regulation of *ompF* mRNA, as well as DsrA sRNA regulation of *hns* mRNA, *Cc* Hfq is able to complement the in vivo annealing effect of the endogenous *Ec* Hfq protein (Fig. 6 *C* and *D*). However, *Cc* Hfq is unable to support annealing of RydC and *cfa* mRNA (Fig. 7*A*). This apparent specificity in annealing activity likely reflects poor binding of *Cc* Hfq to RydC (Fig. 4). In line with these observations, previous work has suggested that the C-terminal tails of *Salmonella* Hfq are important for facilitating formation of the RydC:*cfa*:Hfq ternary complex (41).

**Fig. 8.**
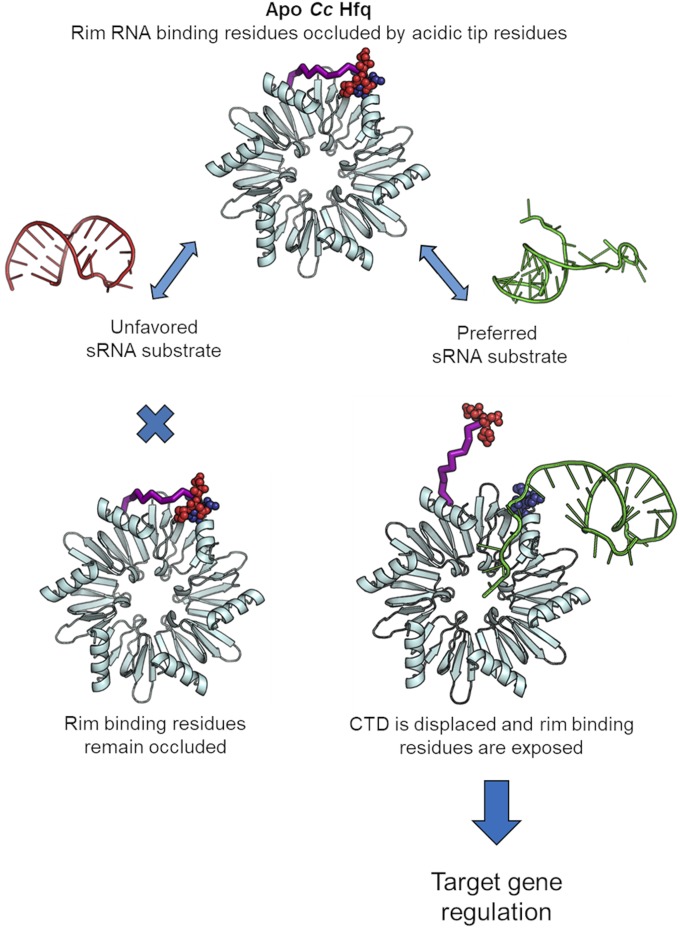
Schematic summary of the role of the *Cc* Hfq CTD. (*Upper*) Apo *Cc* Hfq is shown in cyan. For clarity, a single CTD only is shown as purple ribbon for the linker region and as red spheres for the acidic tip residues. Positive core rim residues are shown as dark-blue spheres, spatially occluded by the acidic tip residues. When a suboptimal sRNA substrate is encountered, the positive rim residues remain occluded by the CTD and RNA binding is unfavored. However, when a preferred sRNA substrate is encountered, the CTD is displaced and the sRNA is able to engage with the positive rim residues, ultimately leading to sRNA binding and target gene regulation.

Our structural, functional, and computational results show how the short CTD of *Cc* Hfq provides specificity for RNA binding and annealing. The role of the Hfq CTD is depicted schematically in Fig. 8. We have demonstrated that although *Cc* Hfq is able to bind RNA in vitro with an affinity close to that of *Ec* Hfq, the RNA annealing activity of the *C. crescentus* protein is greatly reduced. Through the construction of truncated and chimeric proteins, we have shown that the increased inhibition of RNA annealing can be attributed to the acidic residues at the tip of the shorter CTD in *Cc* Hfq. There is an abundance of potential nucleic acid substrates for Hfq in the cell (58). As RNA chaperones with broad substrate specificities, Hfq proteins across the bacterial kingdom seem to be under similar evolutionary pressures to balance electrostatic interactions to RNAs at the basic rims, necessary for RNA annealing and restructuring (15, 16, 59, 60), with gating access to these positively charged patches to avoid sequestration by excess off-target nucleic acids present in the cell. This work unveils a fascinating mechanism of autoinhibition of the conserved Hfq protein that occurs in divergent organisms.

## Materials and Methods

### Crystallization and X-Ray Data Collection.

*Cc* Hfq purified from the N-terminal GST tagged construct was concentrated to 7 mg/mL, and crystals were grown via hanging-drop vapor diffusion by adding an equal volume of crystallization buffer (0.2 M sodium thiocyanate, 17% PEG 3350) to the protein sample. Crystals were harvested using 25% glycerol as a cryoprotectant and then flash-frozen in liquid nitrogen. X-ray data were collected at Diamond Light Source, beamline I03.

### Crystal Structure Determination.

The structure was solved by molecular replacement with PHASER (61) using *Ec* Hfq core [Protein Data Bank (PDB) ID code 4PNO] as a search model. Initial space group assignment and molecular replacement efforts were hampered by the presence of translational noncrystallographic symmetry. PHASER indicated the presence of two nonorigin peaks of 71% and 41% of the origin peak. Ultimately a translational correction was applied, and four copies of the Hfq hexamer were correctly placed in the asymmetric unit of the X-ray data processed in the P4_3_ space group. Following refinement with REFMAC (62) electron density corresponding to the CTD was apparent for several protomers, and the amino acids for this region were modeled manually using Coot (63). Data collection and refinement statistics are summarized in Table 1. The coordinates and structure factors have been deposited in the PDB (ID code 6GWK).

**Table 1.** X-ray data collection and refinement statistics

Parameters/statistics	*Cc* Hfq – 6GWK
Wavelength	
Resolution range	70.35–2.15 (2.227–2.15)
Space group	P4_3_
Unit cell	97.32, 97.32, 203.64–90, 90, 90
Total reflections	168,042 (16,595)
Unique reflections	82,963 (10,091)
Multiplicity	2.0 (1.9)
Completeness, %	97.42 (99.06)
Mean *I*/sigma, *I*	9.44 (0.46)
Wilson B-factor	36.75
*R*_merge_	0.1228 (0.6281)
*R*_meas_	0.1548 (0.823)
*R*_pim_	0.0932 (0.5247)
CC1/2	0.99 (0.618)
CC*	0.998 (0.874)
Reflections used in refinement	99,756 (10,091)
Reflection used for *R*_free_	4,978 (526)
*R*_work_	0.2858
*R*_free_	0.3096
No. of nonhydrogen atoms	12,914
macromolecules	12,804
Solvent	110
Protein residues	1,651
rmsd, bonds	0.003
rmsd, angles	0.54
Ramachandran favored, %	95.18
Ramachandran allowed, %	4.82
Ramachandran outliers, %	0.00
Rotamer outliers, %	0.28
Clashscore	7.61
Average B-factor	54.02
Macromolecules	54.18
Solvent	35.90

Statistics for the highest-resolution shell are in parentheses. CC*, crystallographic refinement statistic.

### Nucleic Acid Preparation.

The sequences of RNA substrates are listed in *SI Appendix*, Table S2. Synthetic target RNAs, the molecular beacon (43), and FAM-labeled A18 (A18-FAM) have been described previously (42). The RNAs R157, R133, ChvR, ChiX, DsrA, RybB, RydC, *cfa*, and *hns* were transcribed in vitro as described previously (56).

### RNA Binding.

The affinities of all Hfq variants for ∼1 nM ^32^P-labeled sRNAs at 30 °C in reaction buffer (34 mM Tris·HCl pH 7.5, 50 mM NaCl, 50 mM KCl, 50 mM NH_4_Cl, 11.4 mM EDTA, 12% glycerol, 0.005% bromophenol blue, 0.005% xylene cyanol FF) for 30 min were measured by native gel EMSAs in 1× TBE (89 mM Tris, 89 mM boric acid, 2 mM EDTA, pH 7.6) as described previously (56), except for *Ec* Hfq complexes with *C. crescentus* sRNAs, which were better resolved in 1× Tris-glycine running buffer (25 mM Tris, 192 mM glycine, pH 8.3). The fraction of ^32^P-labeled sRNA bound to one or more Hfq hexamers, after background subtraction, was fit to a two-state binding isotherm, ƒ_B_ = [Hfq_6_]^*n*^/([Hfq_6_]^*n*^ + *K*_d_^*n*^), in which *n* is the Hill coefficient or steepness of the binding transition. ^32^P-sRNA traveling between the free sRNA and sRNA-Hfq complex was quantified (*SI Appendix*, Fig. S8) and treated as a separate binding transition if this fraction exceeded 10%. In this case, the fraction of tight complexes was fit using ƒ_B_ = ([Hfq_6_]^2^/*K*_d1_*K*_d2_)^*n*^/{1 + ([Hfq_6_]/*K*_d1_)^*n*^ + ([Hfq_6_]^2^/*K*_d1_*K*_d2_)^*n*^}, in which *K*_d1_ is the binding constant for the dissociated complexes and *K*_d2_ is the binding constant for the tight complexes. Binding constants for A18-FAM (5 nM) were measured in 1× TNK buffer (10 mM Tris⋅HCl pH 7.5, 50 mM NaCl, 50 mM KCl) at 30 °C by FAM fluorescence anisotropy as described previously (39).

### RNA Oligomer Annealing.

The kinetics of RNA annealing were monitored by stopped-flow fluorescence spectroscopy as described previously (43, 64) with modifications indicated below. All reactions were carried out at 30 °C in 1× TNK buffer. In the reactions, Hfq protein (0–200 nM) was preincubated for 3 min at 30 °C with target RNAs (Target, Target-A18, or Target-U6, 100 nM), after which molecular beacon was added (50 nM). Annealing progress curves were fit to single- or double-exponential rate equations. Annealing rates relative to the absence of Hfq were calculated as *k*_rel_ = (Hfq *k*_obs_)/(basal *k*_obs_), where Hfq *k*_obs_ is the observed annealing rate constant for a certain target RNA to beacon by a given Hfq at a given concentration and basal *k*_obs_ is the observed annealing rate constant for the same target RNA to beacon in the absence of Hfq. The effect of the CTD was estimated from the ratio of the observed annealing rate, Hfq *k*_obs_, for each Hfq derivative (Ec65, EcCc, and Cc78) to *k*_obs_ in the presence of the parental *Ec* Hfq or *Cc* Hfq protein. Finally, the relative yield *Y* of annealed molecular beacon was calculated from *Y*_rel_ = Hfq *Y*/basal *Y*, where Hfq *Y* is the yield of annealed beacon by a given species and concentration of Hfq and basal *Y* is the yield of annealed beacon with the same target RNA in the absence of Hfq.

### In Vivo Annealing Activity Assays.

Sequences of all oligonucleotides used in this study are listed in *SI Appendix*, Table S3. All bacterial strains used in this study are summarized in *SI Appendix*, Table S4.

Mutant derivatives of *E. coli* MC4100 were constructed by the λRed recombinase one-step inactivation method. To delete or truncate *Ec hfq*, DNA fragments [polymerase chain reaction (PCR) amplification using KFO-0093/KFO-0094 (∆*hfq*) or KFO-0656/KFO-0094 (*Ec*65) on pKD4] were transformed into cells carrying the pKD46 helper plasmid. Accordingly, the *Ec hfq* coding sequence was replaced by transformation of a DNA fragment generated by overlapping PCR (*Cc* Hfq: fragment 1, KFO-0499/KFO-0522 on *C. crescentus* gDNA; fragment 2, KFO-0521/KFO-0094 on pKD4; overlapping PCR KFO-0505/KFO-0506 on fragment1/fragment2). Integration was confirmed by PCR (KFO-0096/KFO-0097); phage P1 transduction (using standard protocols) was used to transfer chromosomal modifications to a fresh wild-type background. To eliminate the KanR cassette of λRed-derived mutants, cells were transformed with the FLP recombinase expression plasmid pCP20.

Unless stated otherwise, *E. coli* were grown aerobically in Luria broth (LB) medium at 37 °C. Where appropriate, liquid and solid media were supplemented with antibiotics at the following concentrations: 100 μg/mL ampicillin, 50 μg/mL kanamycin, and 20 μg/mL chloramphenicol. l-arabinose was added at 0.01% (final concentration) to induce sRNA expression from pP_BAD_ constructs.

For RNA preparation, total bacterial samples were collected, mixed with 0.2 volume of stop-mix (95% ethanol and 5% phenol, vol/vol) and snap-frozen in liquid nitrogen. RNA was isolated using the “hot phenol” method (65). For sRNA and mRNA detection, 5 or 10 μg of total RNA was separated on 7 M urea 6–8% polyacrylamide gels and electroblotted. Membranes were hybridized with gene-specific 5′ end-labeled DNA oligonucleotides at 42 °C in Roti-Hybri-Quick hybridization solution (Roth), and washed in three subsequent steps with SSC wash buffers supplemented with 0.1% SDS (5×/1×/0.5× SSC or 2×/1×/0.5× SSC). RybB sRNA, DsrA sRNA, RydC sRNA, *gfp* mRNA, and 5S rRNA were detected by oligonucleotides KFO-0797, KFO-0701, KFO-0705, KFO-0826, and KFO-0796, respectively. A riboprobe to detect *hfq* mRNA was generated by T7 in vitro transcription of ∼200 ng of template DNA (amplified on *E. coli* gDNA with KFO-0852/KFO-0853) in the presence of [^32^P]‐α‐UTP with the Ambion MAXIscript kit (Thermo Fisher Scientific) according to the manufacturer’s recommendations.

## Supplementary Material

Supplementary File
