# Supplementary material for: Caulobacter crescentus Hfq structure reveals a conserved mechanism of RNA annealing regulation
Source: Proc Natl Acad Sci U S A. 2019 May 10;116(22):10978–87. doi: 10.1073/pnas.1814428116 (PMC6561178; doi:10.1073/pnas.1814428116)
Supplement: Supplementary File [file pnas.1814428116.sapp.pdf]

Supplementary Materials and Methods

Hfq purification

Untagged *E. coli* Hfq102 (*Ec* Hfq) and Hfq65 (*Ec*65) were over-expressed in *E. coli* BL21(DE3) *Δhfq::cat-sacB* cells grown in 1 L LB-Miller media (10 g/L tryptone, 10 g/L NaCl, 5 g/L yeast extract) supplemented with 100 µg/mL ampicillin. Plasmids for over-expression of mutant Hfq proteins were created by site-directed mutagenesis of pET21b-Hfq (1). The purification method has been previously described (2). In brief, resuspended cell lysates of Hfq102 were clarified by heat denaturation and untagged Hfq was purified via Ni^{2+}-affinity. Lysates of Hfq65 variants were further clarified by ammonium sulfate precipitation after heat treatment, and the protein purified by hydrophobic interaction chromatography. Finally, both Hfq variants were purified by cation-exchange chromatography to remove nucleic acids.

Full-length wildtype *C. crescentus* Hfq (*Cc* Hfq), was expressed as either an N-terminal GST fusion protein (pGEX-6p1-*Cc* Hfq) (used for crystallographic studies) or as an untagged protein (petDUET1-*Cc* Hfq) (used for biochemical assays). Chimeric *EcCc* Hfq was expressed as an untagged protein (petDUET1-*EcCc* Hfq). *Cc*78 was expressed with a self-cleavable intein tag (Intein-*Cc* Hfq78). Plasmid pKF491-8 was constructed by Gibson assembly of a DNA fragment encoding truncated *C. crescentus hfq* (PCR amplification from *C. crescentus* NA1000 gDNA using KFO-0561/KFO-0696) and the pTYB11 backbone (PCR amplification using KFO-0798/KFO-0799).

Protein was expressed from all constructs by growing at 37°C in *E. coli* BL21(DE3) cells in 2L of 2xYT media supplemented with 100 µg/ml ampicillin. Over-expression was induced at OD_{600} of 0.4 by addition of 1 mM IPTG, at which point the temperature was lowered to 18 °C and growth continued for a further 16 hours. Cells were harvested by ultracentrifugation and stored at -80 °C prior to protein purification. For protein purification, the harvested cells were thawed, lysed by two

www.pnas.org/cgi/doi/10.1073/pnas.1814428116

passages through an Emulsiflex (Avestin) at 500 bar, and cell lysate was clarified by centrifugation at 30,000 x g for 30 minutes. The clarified lysate was loaded either onto a 5 ml glutathione sepharose column (for pGEX-6p1-Cc Hfq) in GST-buffer A (20 mM Tris, pH 7.5, 200 mM NaCl) and following washing with GST-buffer A was eluted with 15 ml of GST-buffer B (20 mM Tris, pH 7.5, 200 mM NaCl, 50 mM reduced glutathione). The eluate was then incubated overnight at 4°C with 1:50 molar concentration of PreScission protease (GE life science) to cleave the GST tag. For petDUET1-*Cc* Hfq and *EcCc* Hfq, the lysate was loaded onto a 5 ml Hi-trap Q column (GE) in Q-buffer A (20 mM Tris, pH 7.5) and Hfq was eluted in a linear gradient to 100 % Q-buffer B (20 mM Tris pH 7.5, 1M NaCl). For intein- CC Hfq78 the lysate was loaded on to a 5ml chitin resin column in GST buffer A, and following washing with the same buffer the column was flushed with 3 column volumes of GST buffer A supplemented with 40 mM DTT, then the column was capped and left for 48 hours at 4°C to induce cleavage of the intein tag. The liberated Cc78 Hfq was subsequently eluted with 3 column volumes of GST buffer A supplemented with 40 mM DTT. For all protein samples, fractions containing Hfq were diluted four-fold in Q-buffer A, and applied to a 5 ml Heparin column, and again, Hfq was eluted in a linear gradient to 100 % Q-buffer B. Finally, fractions containing Hfq were concentrated and loaded onto a Superdex 200 gel filtration column. Details of all DNA oligonucleotides, bacterial strains and plasmids used in this study are summarised in Supplementary tables 3-5.

Computational modelling of the disordered termini

Rosetta FloppyTail was used to model the disordered termini of *C. crescentus* Hfq. Our approach was based on the original FloppyTail method (3) and modified as previously described (4). Before FloppyTail modelling, the ordered core (residues 9-67) were extracted from chain B of the deposited crystal structure (PDB ID 6GWK), the N- and C-termini were initialized in an extended β-strand conformation, and these models were energy-minimized using Rosetta FastRelax (5) with harmonic restraints to the initial backbone positions limiting motion to less than 1 Å backbone

RMSD. FloppyTail simulations were then run on this input to generate hypothetical, low-energy conformations of the disordered regions. Briefly, FloppyTail is a Monte Carlo method that is primarily composed of random sampling of backbone and side-chain dihedral angles, with occasional gradient-based energy minimization. In a single simulation, generating one possible model, ~500 of these moves are attempted per residue. Typically, 30,000 models are generated, but only the lowest 1% by energy are retained for analysis.

We evaluated pairwise residue–residue interactions using PyRosetta (6). Energies were determined using the *REF2015* energy function (7) which captures van der Waals, solvation, hydrogen bonding, and electrostatic interactions. Only stable pairwise interactions ($E < -2.0$ REU) were considered in our analysis. We determined the probability for a residue to interact with any acidic CTD residue and the average energy of such an interaction. To compute averages and standard deviations we used to bootstrapping, resampling the low-energy models 100 times. Our analysis was previously described (4).

Comparison of the disordered termini models to crystal structures

We recognize that crystal structures may not accurately capture the conformations of disordered protein regions. Nonetheless, we were interested in comparing the CTD conformations in the FloppyTail models to those observed in the crystal structures. From the crystal structures, we chose the subunit with the most resolved residues for further comparison (chain E from PDB ID 6GWK). Each subunit from each model was aligned using to the reference subunit from the crystal structure using the McLachlan algorithm (as implemented in the program ProFit (version 3.1) (8). Alignments were done over the Sm-like domain (residues 6–67) and Cα RMSDs were computed over the Sm-like domain and CTD (residues 6–87 for *C. crescentus*, 6–76 for the C2 chimera, and 6–75 for the P2 chimera).

Contact analysis

In addition to computing Cα RMSDs, we analyzed residue–residue interactions, between residues in the CTD and core, in both crystal structures and models. We used PyRosetta, evaluating interactions with the REF2015 energy function, and determined residue pairs with the NeighborhoodResidueSelector using a 10 Å cutoff.

Experimental details relating to in vitro RNA annealing assays

In annealing experiments presented in this manuscript, the Hfq variants were first incubated with target RNAs before being rapidly mixed with molecular beacon to begin the annealing measurements. In past experiments, however, Hfq was instead pre-incubated with molecular beacon (2, 4). Intriguingly this reversed order of addition gives rise to three differences with respect to previously collected data. First, annealing data for Target-A18 is simpler and can be fit by single- or double-exponential equations, rather than triple-exponential equations, since the fastest phase has been eliminated. Secondly, *Ec*65 is unable to anneal Target-A18 to molecular beacon at any ratio of Hfq:beacon. Thirdly, in previous experiments *Ec* Hfq increased the yield of annealed molecular beacon more than *Ec*65 (2), however the reverse is true for most cases when Hfq is first incubated with the target RNA prior to mixing with molecular beacon. Previous reports suggest that Hfq binds sRNAs and mRNAs in a random order. However, it is important to note that in at least some cases, such as the sRNA-mRNA pair MicC-*ompC*, mRNA・Hfq binary complexes may be dead-ends that are unable to proceed to a functional ternary complex (9). A similar effect may be seen *in vitro* with these simple RNA oligomers in which annealing of Target-A18 to beacon by *Ec* Hfq and *Ec*65 is much slower when Target-A18 is allowed to bind to Hfq first, instead of beacon (Figure 4). Additionally, a 2-fold excess of target RNA (100 nM) over beacon concentration (50 nM) is used in these experiments. Therefore, pre-incubation of Hfq with target RNAs may result in the saturation of Hfq・

target binary complexes, and may enable the formation of erroneous target·Hfq·target ternary complexes. The latter, non-complementary ternary complexes, may be more prone to forming with Hfqs lacking inhibitory CTDs (*Ec*65 and *Cc*78), and at low Hfq_6:beacon concentrations.

Identification of Hfq variants by liquid chromatography tandem mass spectrometry

E. coli strains were grown in LB to OD_{600} of 1.0, and harvested by centrifugation. Cells were washed twice with ice-cold PBS and snap-frozen in liquid nitrogen. Bacteria were lysed by thawing in 50mM Hepes with added protease inhibitor (Liquid chromatography tandem mass spectrometry ™ Protease Inhibitor Cocktail), followed by sonication (Branson Ultrasonics). Cell debris was removed by centrifugation (15 min; 4 °C; 16 000 g), and protein concentration was determined by bicinchoninic assay (BCA, ThermoFisher Scientific). Proteome aliquots of 200 µg were reduced with 10 mM DTT (30 min, 37 °C), alkylated with 50 mM iodoacetamide (30 min, RT, in the dark). Samples were diluted four times with 50 mM ammonium bicarbonate prior to trypsin digestion (proteome/enzyme ratio 100:1 w/w) at 37 °C overnight. Resulting peptides were desalted with home-made C18 stage tips (10), vacuum dried to near dryness, and stored at –80 °C.

Liquid chromatography tandem mass spectrometry (LC-MS/MS) was performed on a nano-LC-system (Ultimate 3000 RSLC, Thermo) coupled to a high-resolution quadrupole Time-Of-Flight mass spectrometer (Impact II, Bruker). The nano-LC system was equipped with an Acclaim Pepmap nano-trap column (C18, 100 Å, 100 µm × 2 cm, Thermo) and an Acclaim Pepmap RSLC analytical column (C18, 100 Å, 75 µm × 50 cm, Thermo). The peptide mixture was eluted over a 75 minute nonlinear gradient of 3-50% buffer B (0.1% [v/v] formic acid and 90 % [v/v] acetonitrile) at constant flow rate of 250 nl / min. The column was kept at 50°C in a column oven throughout the run. MS1 spectra were acquired at 3 Hz with a mass range from m/z 200–2000, with the Top 15 most intense peaks selected for MS/MS analysis using an intensity dependent spectra acquisition time between 4 and 16 Hz. Dynamic exclusion duration was set to 30 sec.

Raw data files were converted in MGF-files using the Compass DataAnalysis software 4.4 (Bruker Daltonics) and peak lists were then searched against a concatenated target/decoy version of the Uniprot *Escherichia coli* (strain K12) database (downloaded on 18th December 2018) using X!Tandem (11), MS Amanda (12), and MS-GF+ (13) search engines within SearchGUI (version 3.3.11; 14). Enzyme specificity was set to fully tryptic, with a maximum of 2 missed cleavages. Carbamidomethylation of C (+57.021464 Da) was set as a fixed modification, whereas acetylation of protein N-term (+42.010565 Da), and oxidation of M (+15.994915 Da) were set as variable modifications. MS and MS/MS tolerances were set to 20.0 ppm as MS1 and 0.05 Da, respectively. Peptides and proteins were inferred from the spectrum identification results using PeptideShaker (version 1.16.36; 15), and validated at 1.0% False Discovery Rate (FDR). The MS raw data associated with the present paper are available upon request.

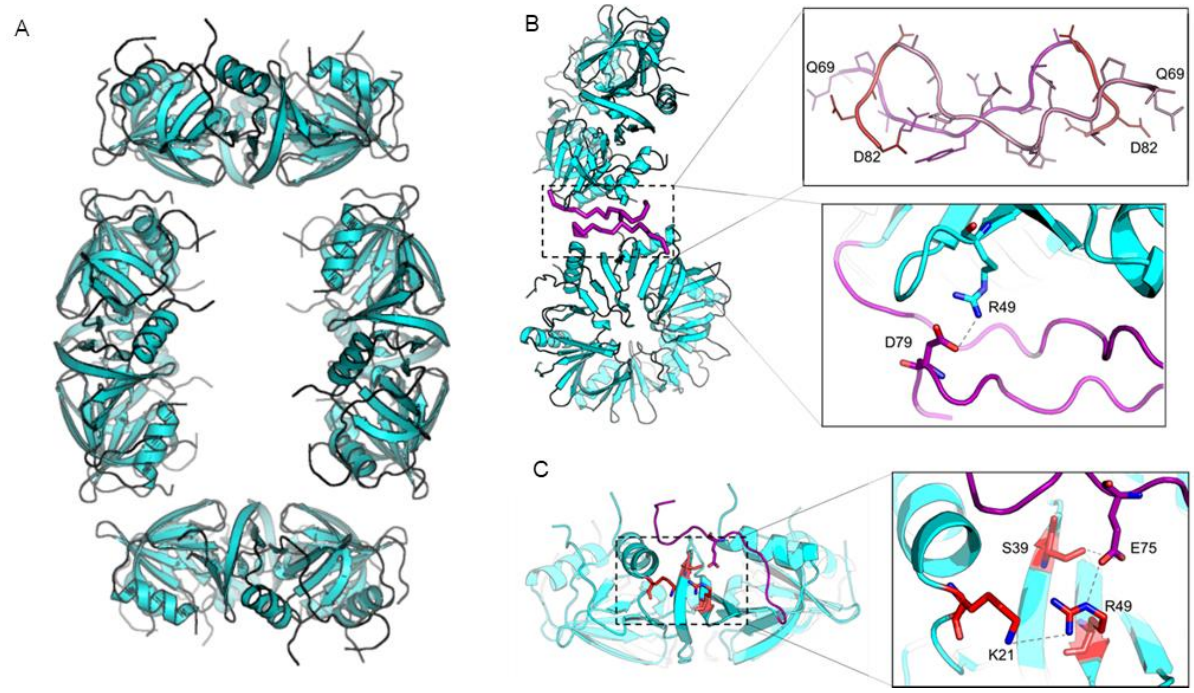

Figure S1. Crystal packing of *C. crescentus* Hfq. (**A**) The contents of a single asymmetric unit from the crystal are shown as cyan cartoon. (**B**) Two neighbouring Hfq hexamers are shown as cyan hexamers, with the C-terminal domains shown as purple ribbons. The top inset shows the adjacent CTD's as ribbons in two shades of purple with sticks, with the acidic tip residues coloured red. The lower inset highlights residues aspartate 79 (purple stick) and arginine 49 (cyan stick) from neighbouring hexamers. (**C**) Left and inset: glutamate 75 from the CTD forms hydrogen bonds with arginine 49 and serine 39 of a neighbouring protomer. Arginine 49 also is within hydrogen bonding distance of lysine 21 from the same protomer. Hydrogen bonds are shown as dashed grey lines.

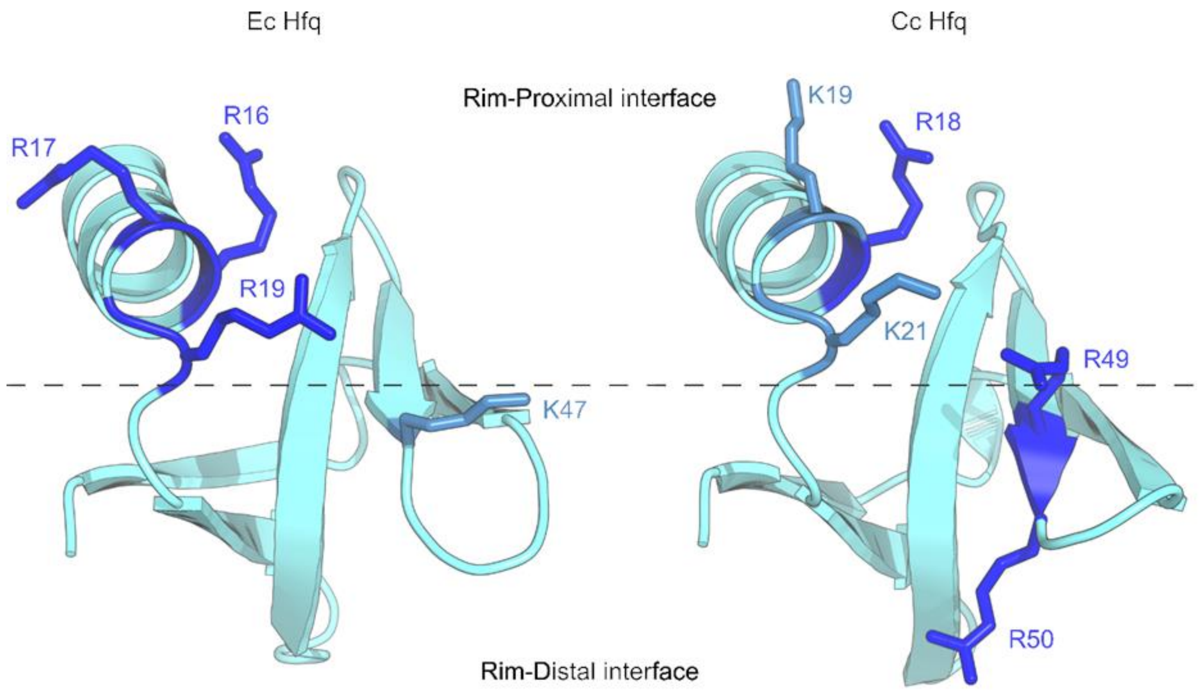

Figure S2. Comparison of basic residue distribution across the rims of *Ec* and *Cc* Hfqs. Side-on view of Hfq monomers shown as cyan cartoons. Surface exposed arginines (royal blue) and lysines (skyblue) at the rim are shown as sticks. Basic residues within the alpha-helix are closer to the proximal face, whereas those within the third beta-sheet are closer to the distal face. NTE and CTD are omitted for clarity.

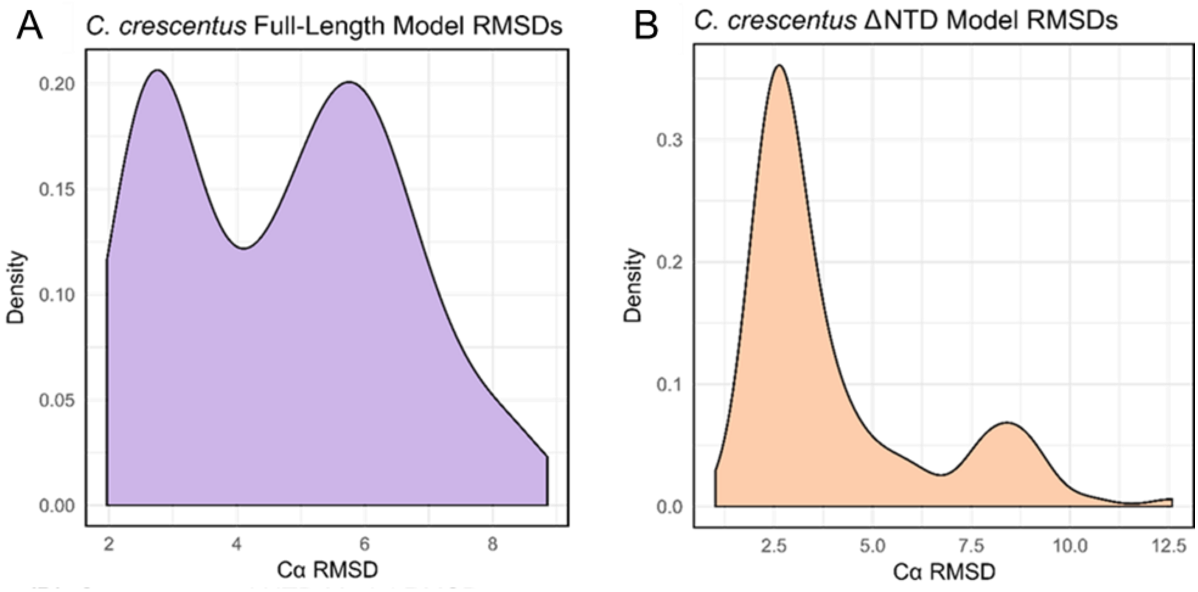

Figure S3. Kernel density estimates of model–crystal Cα RMSD distributions. The alpha carbon coordinates of each subunit from Hfq hexamers modelled with Rosetta Floppy Tail were compared to the coordinates of the subunit with the greatest number of resolved residues from the crystallographic structure. (**A**) Low-energy *C. crescentus* Hfq models have a significant population of structures with low-RMSD. (**B**) N-terminal extension residues were excluded from the simulation to eliminate NTE-CTD interactions. Excluding the NTE during modelling increases the low-RMSD population of models.

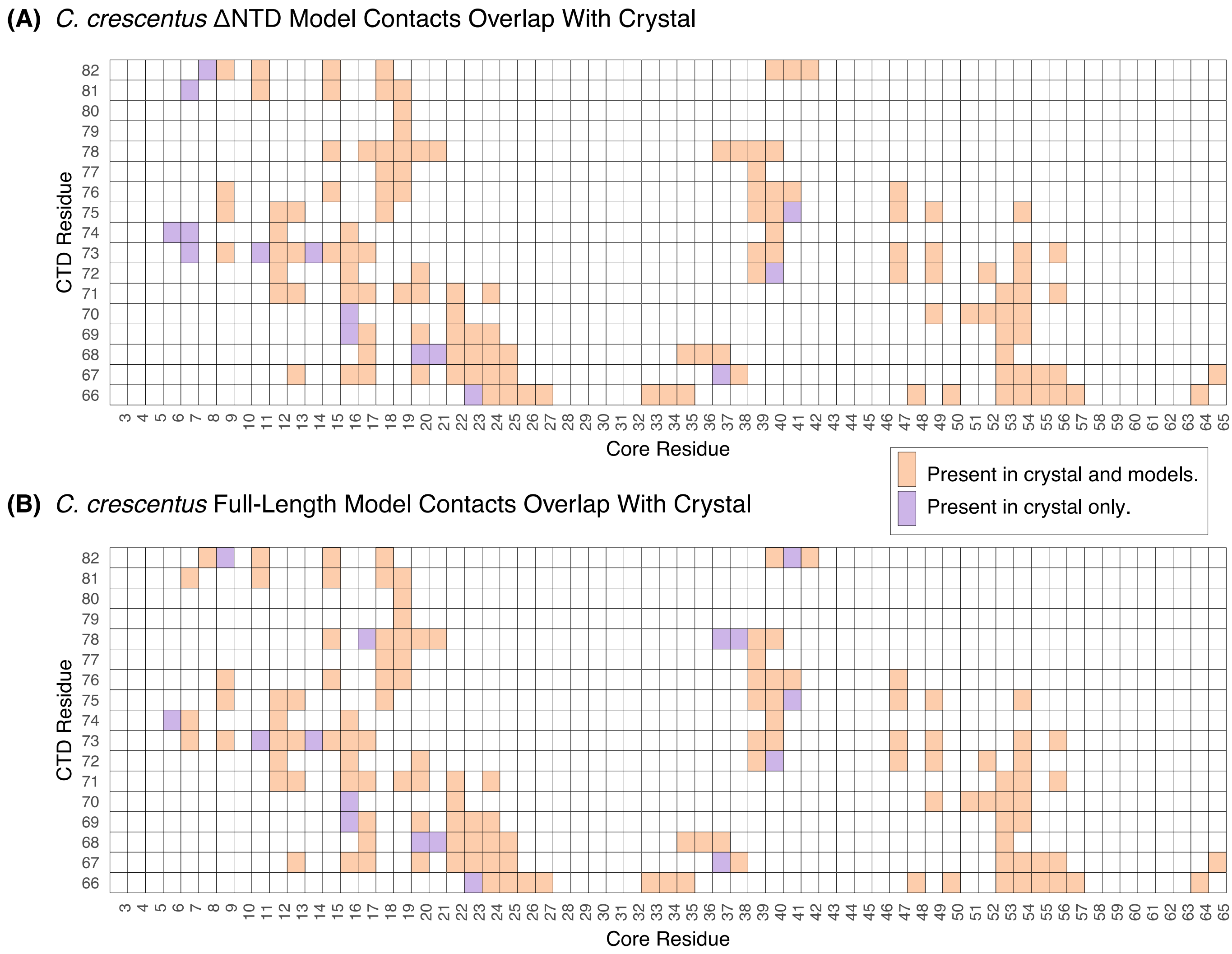

Figure S4. CTD-core contacts present in both the *C. crescentus* Hfq crystal structure and the computational models (orange boxes) or in the crystal structure only (purple boxes). (**A**) The top 20 (by energy) ΔNTE models capture 128/143 crystallographic contacts. (**B**) The top 20 (by energy) full-length models capture 127/143 contacts. Residue pairs with Cβ-Cβ distances less than 10 Å are defined as interacting in the above plots, as described in the supplementary methods.

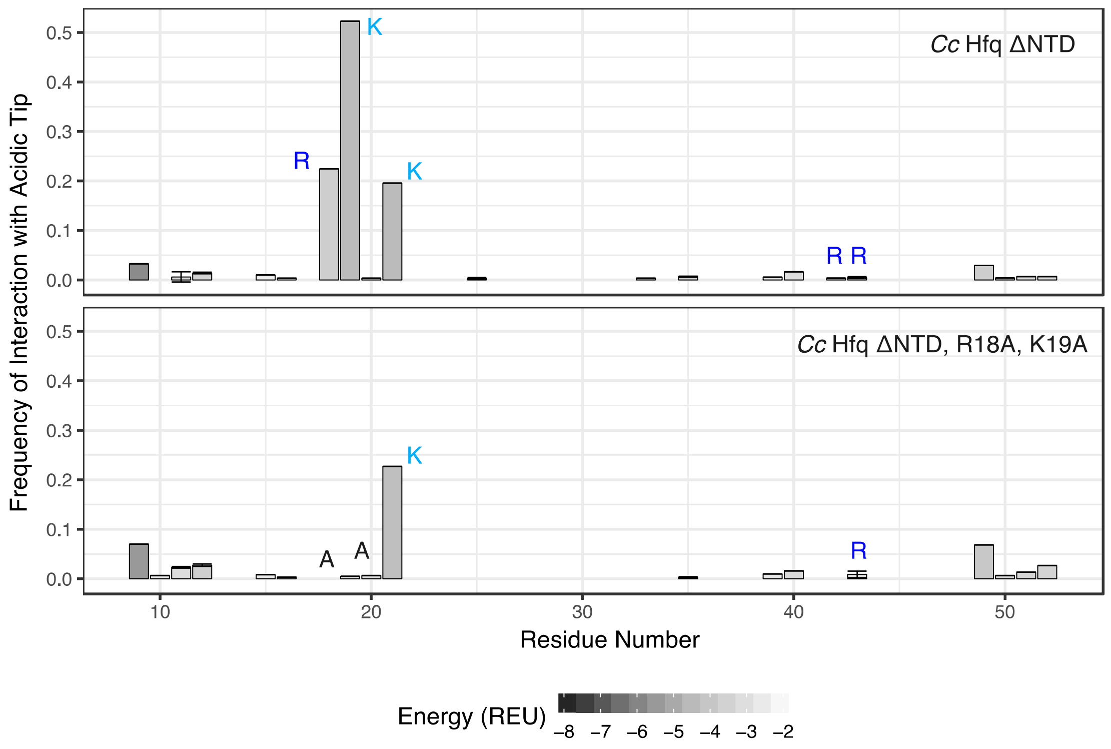

Figure S5. Observed frequency of favourable (E < -2.0 REU) core residue-to-acidic CTD interactions in low-energy FloppyTail models for *Cc* Hfq excluding the N-terminus (top panel) and a variant with two rim residues mutated to alanine (R18A, K19A; bottom panel). Mutating out the positive rim residues obviates interactions *in silico*. Error bars show ± one standard deviation, as computed by bootstrap resampling.

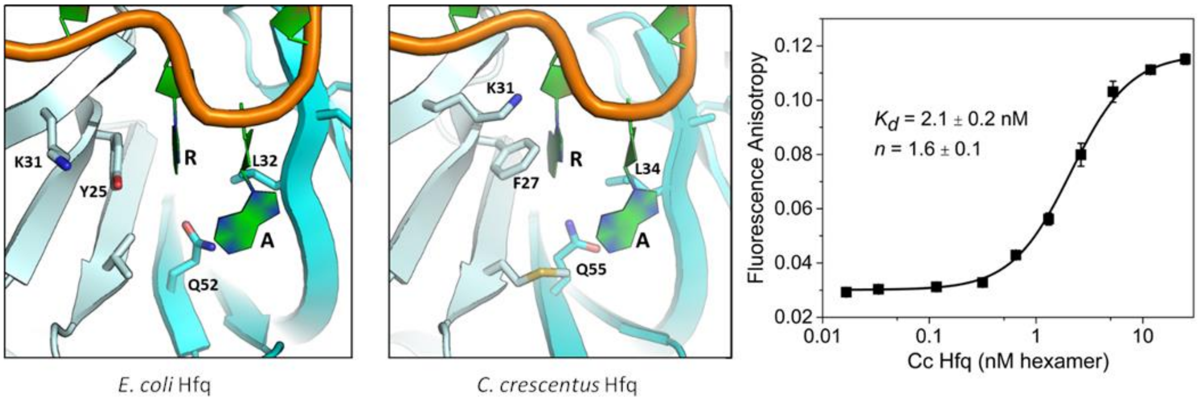

Figure S6. Binding of A18 RNA to *C. crescentus* Hfq. Left; The structure of *Ec* Hfq bound to polyA RNA (PDB 3GIB). Hfq is coloured cyan, with residues in the RNA binding pocket shown as sticks and labelled. RNA is shown as green and orange cartoon, with bases in the R and A binding sites labelled. Middle panel; the RNA from 3GIB docked onto the structure of *Cc* Hfq. Equivalent amino acids as in the left panel are labelled. Right panel; fluorescence anisotropy binding curve for A18-FAM RNA (5 nM) and *Cc* Hfq. Data were fit to a single binding isotherm with Kd = 2.1 nM Hfq_6, ± 0.2.

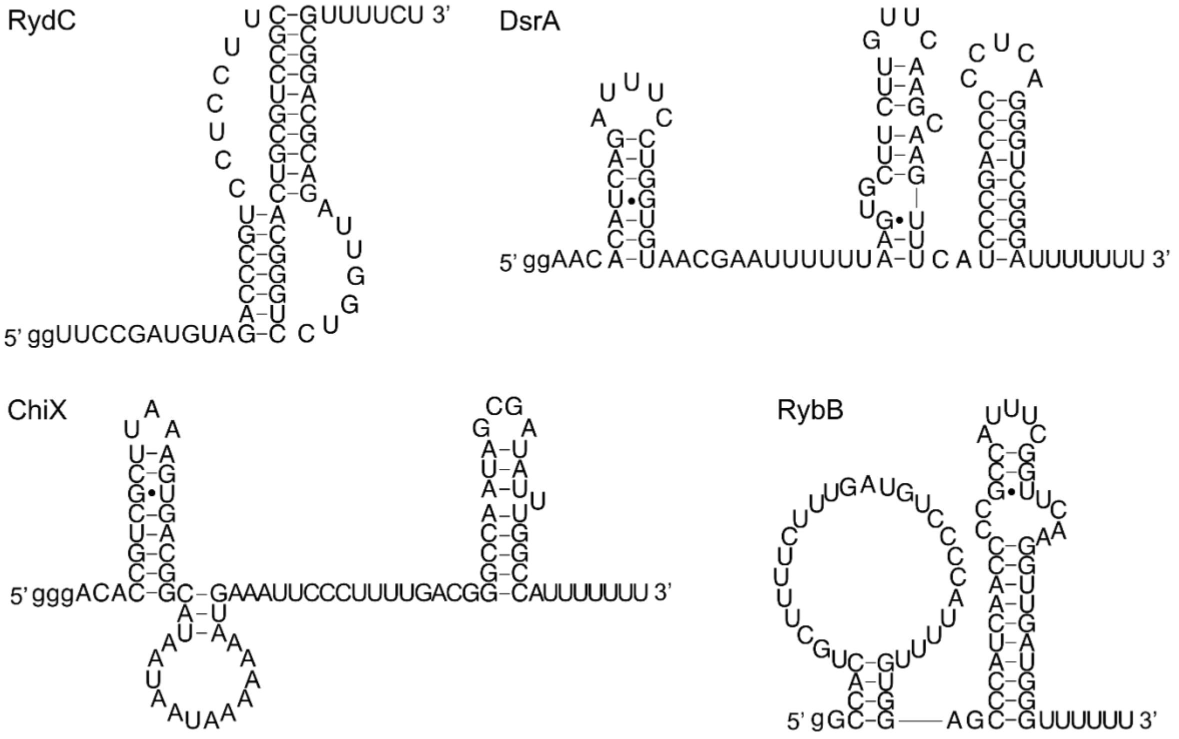

Figure S7. Secondary structures of the four *E. coli* sRNAs used in this study. Structures are based on ribonuclease structure probing (16; Yi Pang, Ph.D. thesis, Johns Hopkins University, 2014) and empirical free energy predictions.

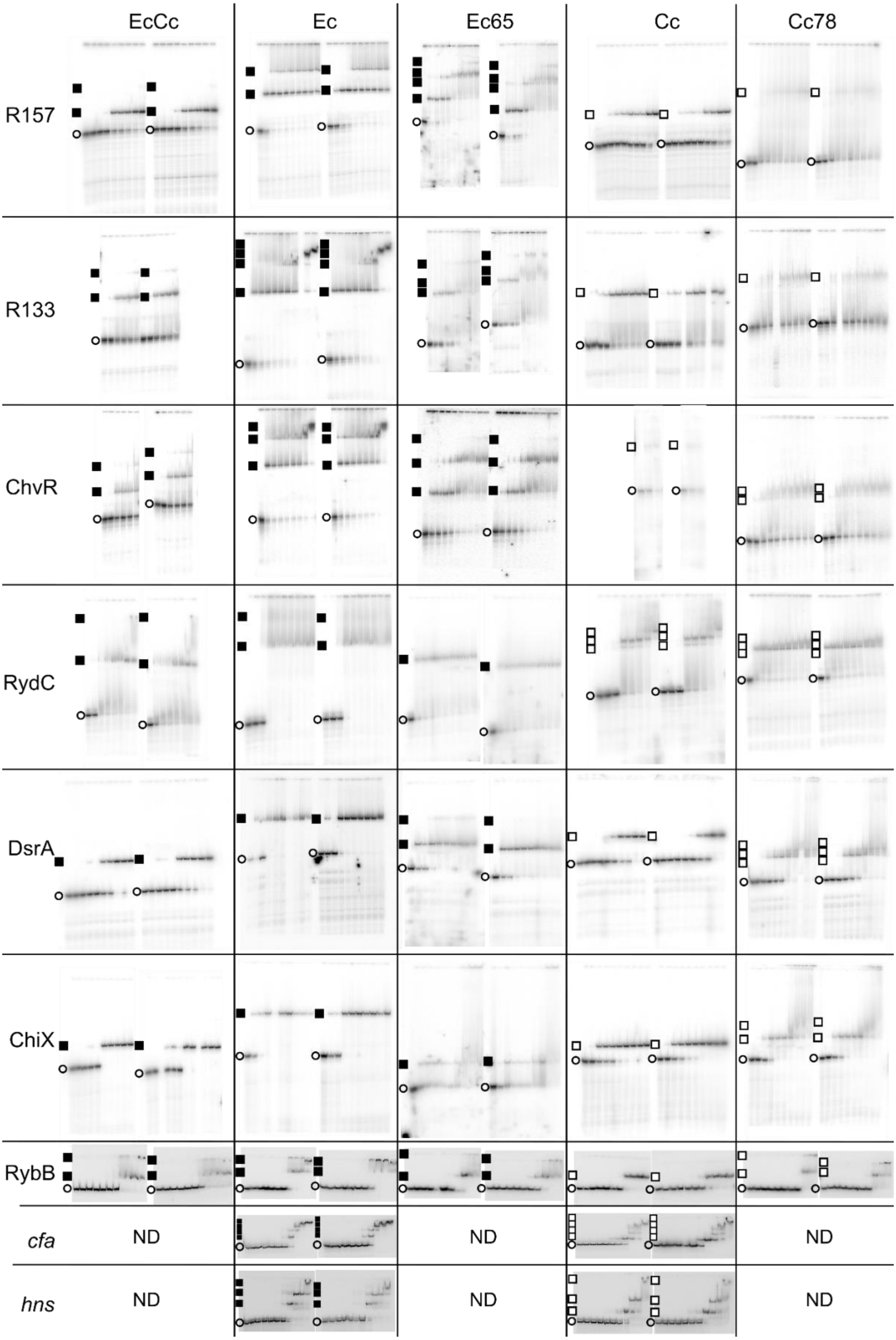

EcCc
Ec
Ec65
Cc
Cc78
R157
R133
ChvR
RydC
DsrA
ChiX
RybB
cfa
hns
ND
ND
ND
ND
ND
ND

Figure S8. Example duplicate gels of *E. coli* and *C. crescentus* Hfq variants binding to sRNAs and mRNAs from both bacteria. Each titration occurs from left to right, increasing protein concentration. Free RNA bands are denoted with an open circle to the left of them. Bound RNA bands are denoted with solid (*E. coli* Hfqs) or open (*C. crescentus* Hfqs) boxes. The number and range of Hfq concentrations for each titration differ (Table S6); these were adjusted to more accurately measure the affinity of the given RNA-Hfq interaction. RybB, *cfa* and *hns* EMSAs were run under lower current during electrophoresis, and therefore these Hfq-RNA complexes do not show as much dissociation during electrophoresis.

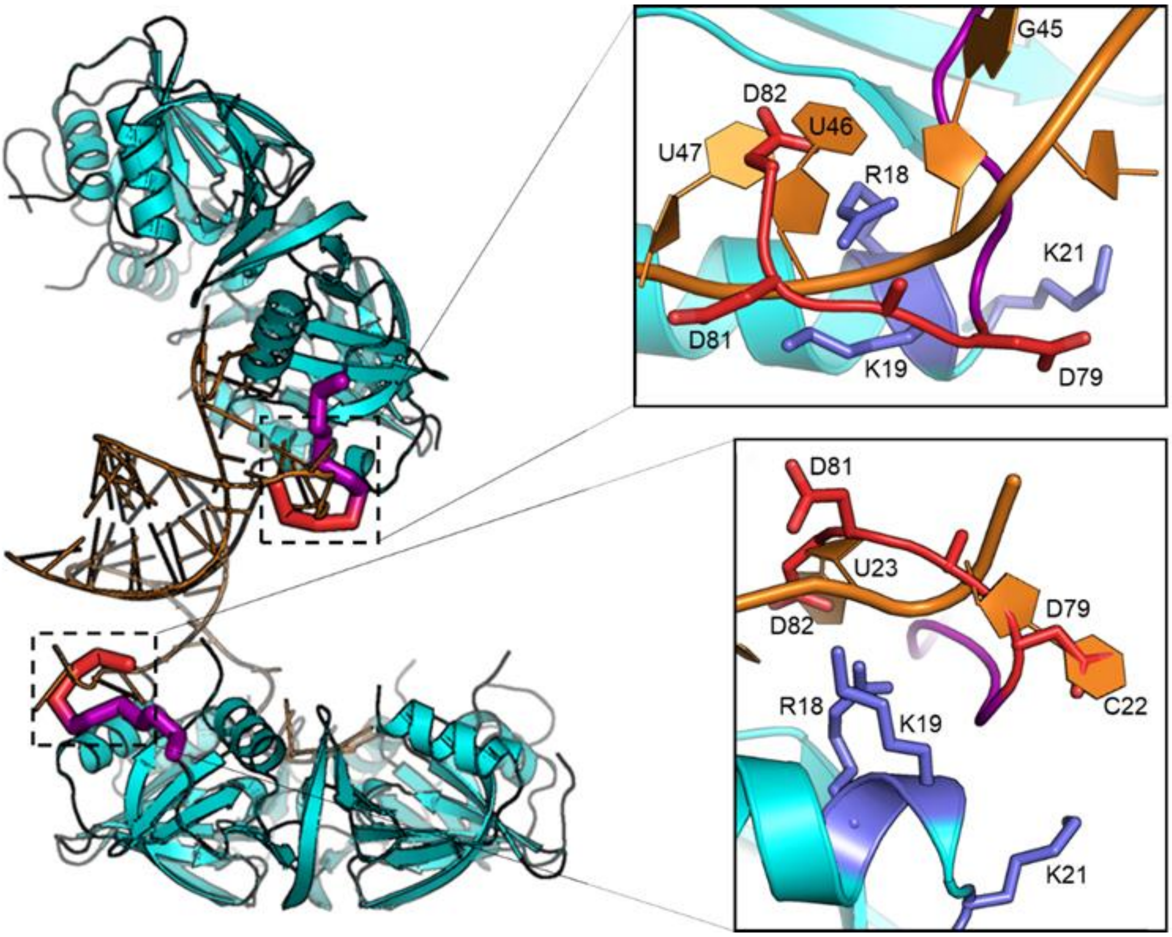

Figure S9. Composite model of *Cc* Hfq bound to RydC. Left: The *Cc* Hfq hexamer (cyan) in place of the *Ec* Hfq hexamer bound to RydC (PDB: 4V2S). RNA is shown as orange cartoon, a single CTD from each hexamer is shown as purple ribbon with acidic tip residues (DADD) in red. Insets show a close up view of steric clashes in the model between RNA and protein residues from within the CTD.

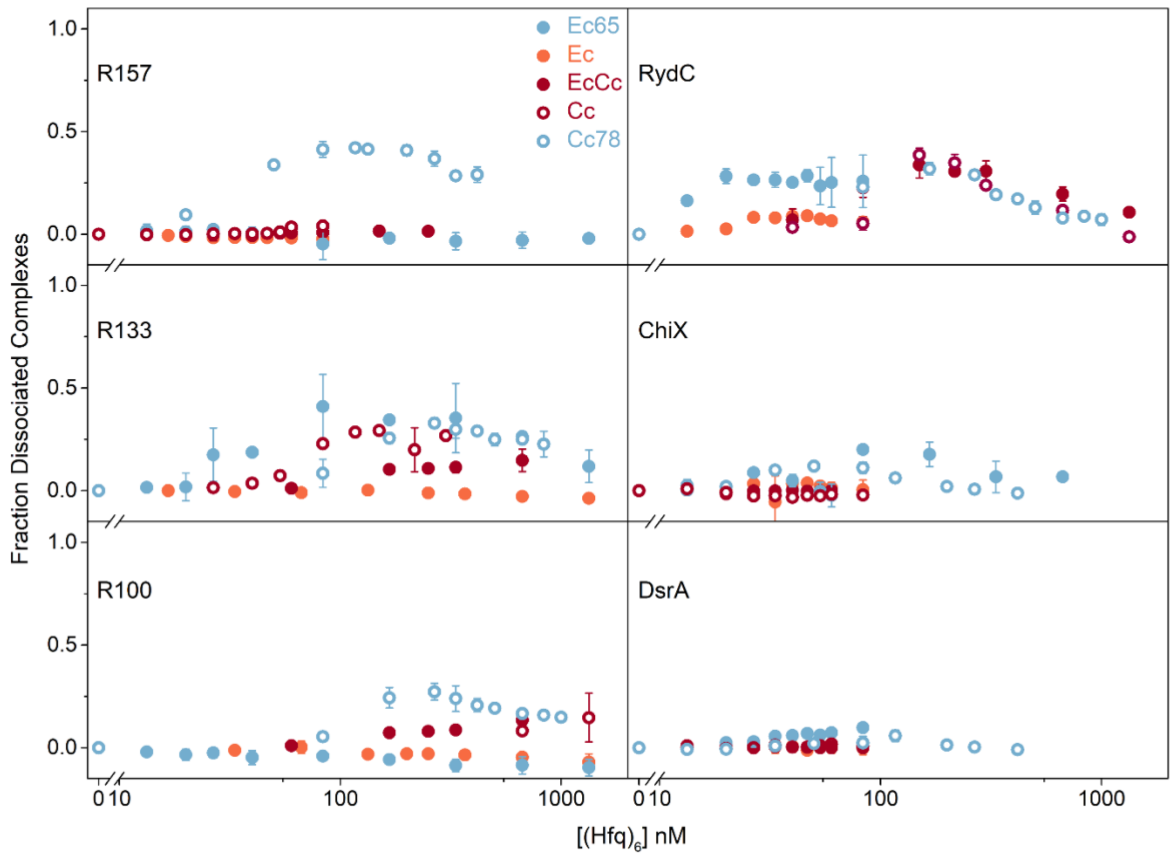

Figure S10. Fraction of weakly bound Hfq-RNA complexes. Complexes which dissociate during electrophoresis migrate between the free sRNA band and the first discrete bound complex. These complexes are not included in the "bound fraction" plotted in Figure 3. Data is shown *E. coli* (solid lines; filled circles) or *C. crescentus* (dotted lines; open circles) Hfqs bearing no acidic CTD tip (light blue), a CTD with a distantly tethered acidic tip residues (orange), or a CTD with a closely tethered acidic tip residues (red). RybB, *cfa* and *hns* EMSAs were run under lower current during electrophoresis, so these Hfq-RNA complexes do not show as much dissociation during electrophoresis (Figure S8) and are not included in this plot.

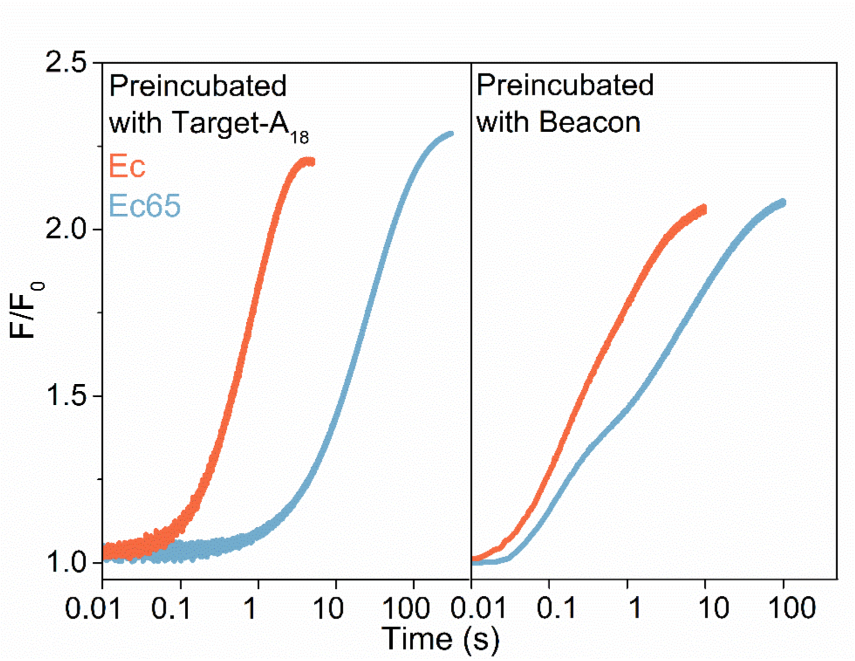

Figure S11. Comparison of *in vitro* RNA annealing yields and rates for different orders of substrate binding. Stopped-flow fluorescence progress curves for annealing of Target-A18 to molecular beacon, by 50 nM of Ec (orange) of Ec65 (light blue) Hfq hexamer. Hfqs were either allowed to pre-bind Target-A18 (left; as done in Figure 5), or beacon (right; 2, 17) before being rapidly mixed with the complementary RNA (beacon or Target-A18 respectively). The reaction kinetics for this pair of RNAs are simpler when Hfq is pre-incubated with Target-A18 first, rather than beacon. Although there is a greater difference in the annealing rates between the two Hfqs when they are pre-incubated with Target-A18, the general trend is conserved between both orders of RNA substrate addition. Progress curves we normalized to the initial fluorescence reading (F_0).

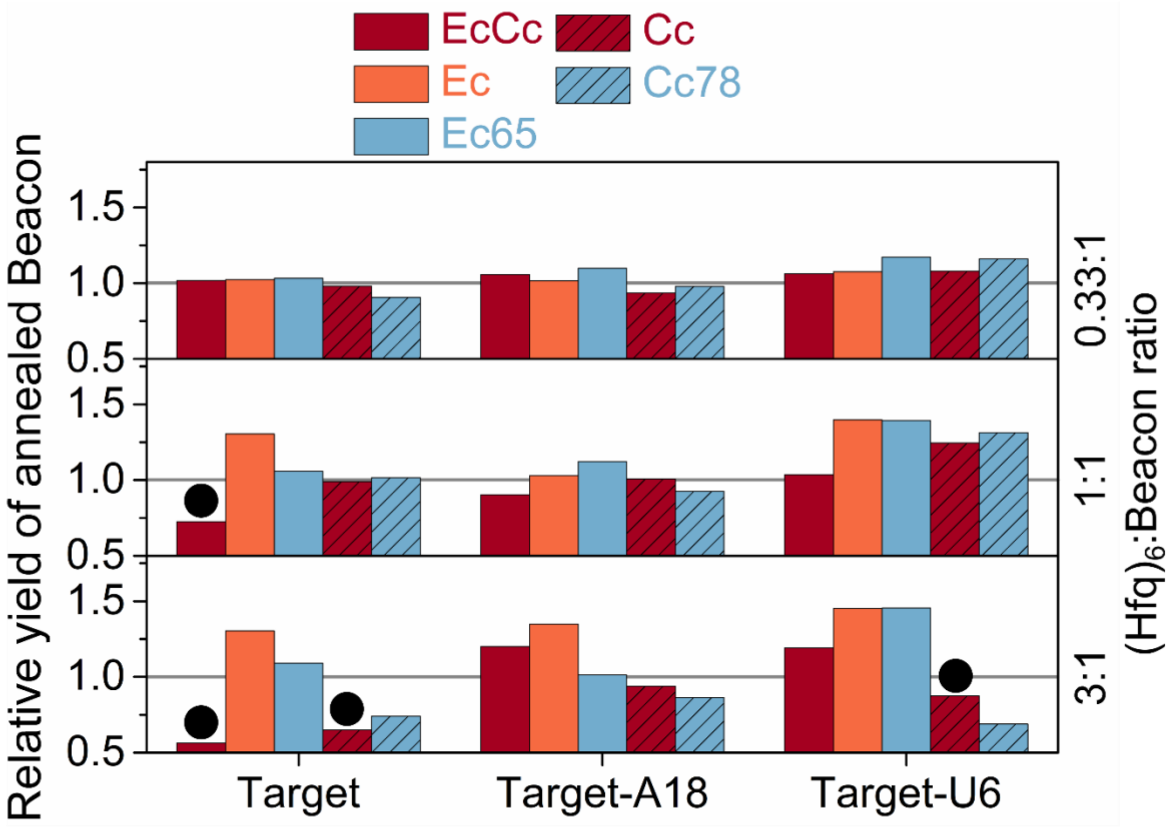

Figure S12. Yield of annealed target•beacon product by different Hfq variants. Relative amount of annealed molecular beacon product generated after a maximum of 500 seconds, for increasing ratios of $(Hfq)_6$:Beacon. Data is shown for *E. coli* (solid bars) or *C. crescentus* (hatched bars) Hfqs bearing no acidic CTD tip (light blue), a CTD with a distantly tethered acidic tip residues (orange), or a CTD with a closely tethered acidic tip residues (red). Annealing reactions which were not complete within 500 s are noted with a black dot overhead. Poor yield can arise from sequestration of beacon and target RNA on separate Hfq hexamers, especially in high protein:RNA ratios, that limits the formation of productive ternary complexes. This is partially overcome by U6 and A18 binding sites for Hfq.

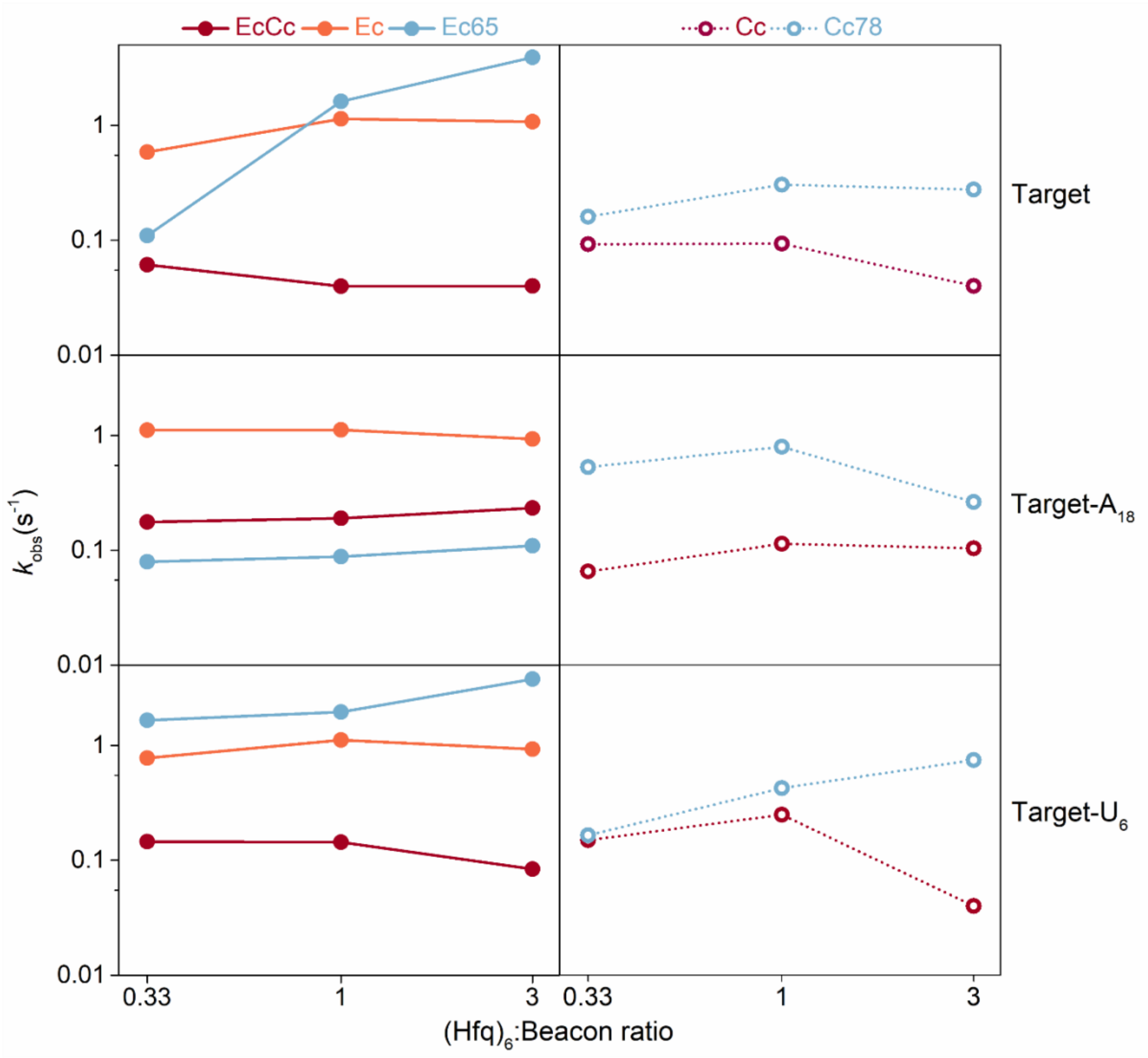

Figure S13. Observed rate constants for the annealing of Target RNAs and Molecular Beacon measured by stopped-flow fluorescence (Fig. 4). Data is shown *E. coli* (solid lines; filled circles) or *C. crescentus* (dotted lines; open circles) Hfqs bearing no acidic CTD tip (light blue), a CTD with a distantly tethered acidic tip residues (orange), or a CTD with a closely tethered acidic tip residues (red). Reactions were carried out with increasing concentrations of *E. coli* and *C. crescentus* Hfq variants. The annealing of Target RNA by the highest concentrations of *Cc* and *EcCc* Hfqs, and the annealing of Target-U6 by the highest concentration of *Cc* Hfq were too slow to be accurately measured and are instead upper estimates.

Figure S14. Expression of Hfq variants in *E. coli* MC4100.

A

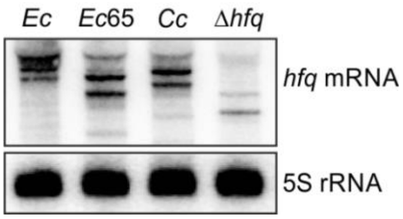

B

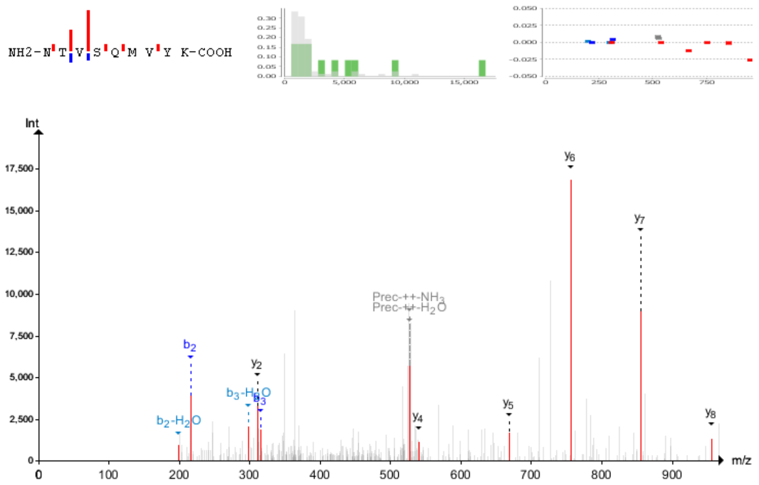

C

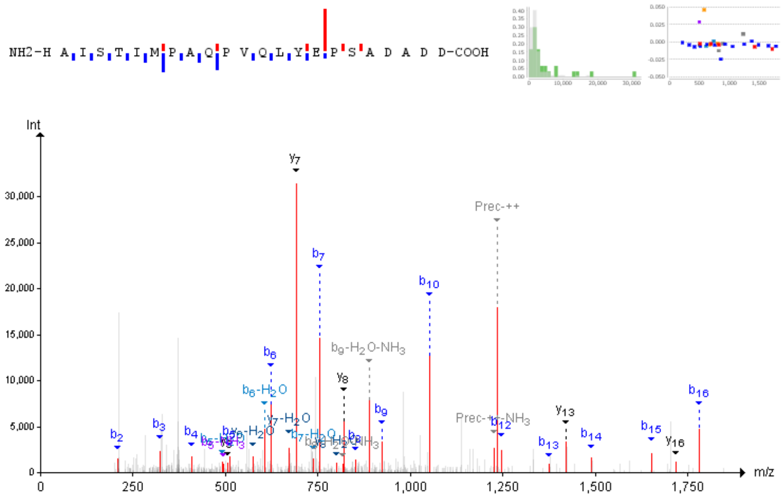

Figure S14. Identification of Hfq by Northern blots and shotgun proteomics.

(A) Northern blot analysis of *hfq* mRNA levels in reporter strains KFS-0191 (*Ec* Hfq), KFS-0705 (*Ec*65 Hfq), KFS-0706 (*Cc* Hfq) and KFS-0708 (*Δ hfq*). A riboprobe recognizing the common 5' UTR of all variants was used to probe the *hfq* transcripts. (B,C) Representative MS/MS annotated spectra obtained by LC-MS/MS analysis used to validate the expression of *Ec* Hfq (in KFS-0191) (**B**) and *Cc* Hfq in KFS-0706 (**C**). Both peptides were identified with 100% confidence using PeptideShaker version 1.16.36 (15). The plots above the spectra show the fragmentation of the peptide (left); the intensity distribution of annotated peaks (green) and non-annotated peaks (grey) with intensity bins on the x-axis and frequency on the y-axis (middle); the standard mass error plot with m/z on the x-axis and mass error (in Da) on the y-axis (right).

Table S1. Equilibrium dissociation constants for Hfq binding to sRNAs and mRNAs.[a]

C. crescentus RNAs

**Hfq**	***Kd* (nM)**					
	R157		R133		ChvR	
Cc	61.2	± 2.1	68.7	± 1.5	>500	
Cc78	349	± 7	242	± 17	256	± 25
Ec	14.3	± 0.1	14.7	± 1.3	17.2	± 1.0
EcCc	55.7	± 2.7	232	± 46	228	± 84
Ec65	6.3	± 1.1	21.6	± 1.3	20.2	± 0.3

	n					
	R157		R133		ChvR	
Cc	4.1	± 0.6	4.6	± 0.4	–[b]	
Cc78	1.3	± 0.3	1.2	± 0.3	1.4	± 0.2
Ec	8.2	± 0.5	1[c]		1.0	
EcCc	5.1	± 1.4	1.8	± 0.5	1.6	± 0.6
Ec65	1.0	± 0.1	1.7	± 0.2	1.6	± 0.1

E. coli RNAs

**Hfq**	***Kd* (nM)**											
	ChiX		DsrA		RydC		RybB		*cfa*		*hns*	
Cc	22.7	± 0.9	47.2	± 1.2	206	± 17	28.5	± 0.0	13.9	± 2.2	17.8	± 0.8
Cc78	32.4	± 0.4	62.2	± 4.4	167	± 11	68.1	± 1.9	–[b]		–[b]	
Ec	20.3	± 0.4	21.1	± 0.3	22.1	± 0.2	21.5	± 2.0	19	± 0.5	19.7	± 0.4
EcCc	35.8	± 0.4	42.5	± 1.0	237	± 34	37.3	± 6.2	–[b]		–[b]	
Ec65	95	± 27	12.6	± 1.9	12.3	± 0.4	27.1	± 0.9	–[b]		–[b]	

	n											
	ChiX		DsrA		RydC		RybB		*cfa*		*hns*	
Cc	4	± 0.6	8.6	± 1.5	2.9	± 0.6	4.6	± 1.2	4.0	± 1.0	3.8	± 1.1
Cc78	3.5	± 0.4	2.5	± 0.4	1.6	± 0.1	5.5	± 0.1	–[b]		–[b]	
Ec	10.1	± 3.1	11.9	± 2.6	13	± 0.8	4.1	± 0.2	2.2	± 0.5	4.4	± 1.0
EcCc	30.2	± 4.4	8.7	± 1.5	1.0		4.5	± 0.9	–[b]		–[b]	
Ec65	1.0		2.4	± 1.2	3.7	± 0.5	3.1	± 0.1	–[b]		–[b]	

[a]K_d and Hill (n) coefficients are the midpoints and gradients, respectively, of two independent experiments at 30 °C in 34 mM Tris-HCl pH 7.5, 50 mM NaCl, 50 mM KCl, 50 mM NH_4Cl, 11.4 mM EDTA, 12 % glycerol, 0.005 % bromophenol blue, 0.005 % xylene cyanol FF). Hfq binding was measured by EMSA (see Figure S8 and Methods). The fraction of tightly bound RNA was fit to two-state cooperative binding isotherms. More elaborate binding models did not produce statistically more reliable results for those reactions that resulted in more than one tightly bound Hfq-RNA complex. Three-state binding models were used to fit binding reactions of Cc78 Hfq with sRNAs R157, R133, and ChvR, or Cc, Cc78, and EcCc Hfq with RydC sRNA. These reactions populate weakly bound complexes at low Hfq concentrations that were treated as a separate bound state in the models (see Methods). The parameters in the table are for the formation of tightly bound complexes.

[b]Not determined.

[c]Binding was not cooperative (n = 1).

Table S2. Sequences of RNA substrates used in this study.

RNA oligomers	
Target	GUGGUCAGUCGAGUGG
Target-U6	GUGGUCAGUCGAGUGGUUUUUU
Target-A18	GUGGUCAGUCGAGUGGAAAAAAAAAAAAAAAAAA
A18-FAM	FAM-AAAAAAAAAAAAAAAAAA
Molecular beacon	FAM-GGUCCCCCACUCGACUCACCACCGGACC-DABCYL
Transcribed RNAs	
R157	gGUGAGGCGCCGAGCGCCUCUGCCCUUCCUGGGCGUUUCCUCCCUAU<br>GACUUUGUAGCCCGGUCUUCAUGGCCGGGCUUUCUUUUU
R133	ggAGCCCUUUCGGGCGUUUCCUCCCAAUGACUCGGCCGCCCUCUUCU<br>CCUGGAGGGCGGCCGUUCUUUU
ChvR	GGCGGGGCCUACAUGUCGCGCAAGUCUCCGGAGGGCCAUUCCGAGGC<br>GGGGCCCGCUCCAGCGUCCUCAUUGGAGCGGGCGUCU
DsrA	gggAACACAUCAGAUUUCCUGGUGUAACGAAUUUUUUAAGUGCUUCU<br>UGCUUAAGCAAGUUUCAUCCCGACCCCCUCAGGGUCGGGAUUUUUUU
ChiX	gggACACCGUCGCUUAAAGUGACGGCAUAAUAAUAAAAAAAUGAAAU<br>UCCUCUUUGACGGGCCAAUAGCGAUAUUGGCCAUUUUUUU
RydC	ggcUUCCGAUGUAGACCCGUAUUCUUCGCCUGUACCACGGGUCGGUUUUAGU<br>ACAGGCGUUUUCUU
RybB	gGCCACUGCUUUUCUUUGAUGUCCCCAUUUUGUGGAGCCCAUCAACCCCGCCA<br>UUUCGGUUCAAGGUUGAUGGGUUUUUU
cfa	gGUUGUUAAAGCUACGAUAAAUAUUAUGUUUUUACGGGGACAGGAUCGUUC<br>CCGACUCACUAUGGAUAGUCAUUUCGGCAAGGGUUCCUCCUUUCCCUCUGUU<br>CUACGUCGGAUUAUAGACUCGCGGUUUUUUCUGCGAGAUUUCUCACAAAGCC<br>CAAAAAGCGUCUACGCUGUUUUAAGGUUCUGAUCACCGACCAGUGAUGGAGA<br>AACU**AUG**AGUUCAUCGUGUAUAGAAGAAGUCAGUGUACCGGAUGACAAC
hns	gAACAAACCACCCCAAUAUAAGUUUGAGAUUACUACA**AUG**AGCGAAGCACUUA<br>AAAUUCUGAACAACAUCCGUACUCUUCGUGCGCAGGCAAGAGAAUGUACACU<br>UGAAACGCUGGAAGAA

[a]Lower-case letters represent non-natural guanosines added to the 5′ end of the sRNA or mRNA to aid *in vitro* transcription. mRNA start codons are shown in bold. The first nucleotise of *cfa* mRNA (adenosine) was replaced with a single guanosine. The RybB sequence was previously used in binding assays (18).

Table S3. Sequences of all DNA oligomers used in this study.

Name	Sequence (5'-3')	Description
ccHFQ pGEX.	GCGGATCCATGTCCGCCGAAAAGAAGC	
ccHFQ pGEX.	GCGGATCCATGTCCGCCGAAAAGAAGC	
ccHFQ duet.F	CGCATATGTCCGCCGAAAAGAAGC	
ccHFQ.R	CGCTCGAGTCAGTCGTCGGCGTCGGC	
EcCc Hfq.F	CGCATATGGCTAAGGGGCAATCTTTAC	
EcCc Hfq.R	GCCGGATCCTCAGTCGTCGGCGTCGGCGCTCGGC TCATAGAGCTGAACCGGGCGAGACGGG	
ccHFQ pGEX.	GCGGATCCATGTCCGCCGAAAAGAAGC	
ccHFQ duet.F	CGCATATGTCCGCCGAAAAGAAGC	
ccHFQ.R	CGCTCGAGTCAGTCGTCGGCGTCGGC	
EcCc Hfq.F	CGCATATGGCTAAGGGGCAATCTTTAC	
EcCc Hfq.R	GCCGGATCCTCAGTCGTCGGCGTCGGCGCTCGGC TCATAGAGCTGAACCGGGCGAGACGGG	
KFO-0093	AAGGTTCAAAGTACAAATAAGCATATAAGGAAA AGAGAGAGTGTAGGCTGGAGCTGCTTC	*E. coli hfq* mutant construction
KFO-0094	AGGATCGCTGGCTCCCCGTGTAAAAAAACAGCCC GAAACCGGTCCATATGAATATCCTCCTTAG	*E. coli hfq* mutant construction
KFO-0096	GCGTGACGAAGTATTACAGG	*E. coli hfq* mutant construction
KFO-0097	GTCTTCCATATCTTTGTCTTGC	*E. coli hfq* mutant construction
KFO-0351	GTTTTTTTTTTAATACGACTCACTATAGGCGGGGC CTACATGTCG	PCR template T7 transcription ChvR
KFO-0353	AGACGCCCGCTCCAATGA	PCR template T7 transcription ChvR
KFO-0499	TCAGAATCGAAAGGTTCAAAGTACAAATAAGCA TATAAGGAAAAGAGAGAATGTCCGCCGAAAAGA AGCAA	*E. coli hfq* mutant construction
KFO-0505	TCAGAATCGAAAGGTTCAAAGTA	*E. coli hfq* mutant construction
KFO-0506	CGGGGAACGCAGGATCG	*E. coli hfq* mutant construction
KFO-0521	CGACGACTGAGTGTAGGCTGGAGCTGCTTC	*E. coli hfq* mutant construction
KFO-0522	CAGCCTACACTCAGTCGTCGGCGTCGGC	*E. coli hfq* mutant construction

KFO-0561	CCCAGGTTGTTGTACAGAACATGTCCGCCGAAAA GAAGC	construction of plasmid pKF491-8
KFO-0563	GTTTTTTTTTTAATACGACTCACTATAGGTGAGGC GCCGAGCGC	PCR template T7 transcription CCNA_R0157
KFO-0564	AAAAAGAAAGCCCGGCCATG	PCR template T7 transcription CCNA_R0157
KFO-0567	GTTTTTTTTTTAATACGACTCACTATAGGAGCCCT TTCGGGCGTTTC	PCR template T7 transcription CCNA_R0133
KFO-0568	AAAAGAACGGCCGCCCTC	PCR template T7 transcription CCNA_R0133
KFO-0656	CGGTCAGCCAGATGGTTTACAAGCACGCGATTTC TACTGTTGTCCCGTCTTAA GTGTAGGCTGGAGCTGCTTC	*E. coli hfq* mutant construction
KFO-0696	CGGATCCCCTTCCTGCAGTCAGGCGCTCGGCTCA TAGAG	construction of plasmid pKF491-8
KFO-0701	TCGTTACACCAGGAAATCTGATGT	Oligo probe to detect Eco *DsrA*
KFO-0705	AGAAAACGCCTGTACTAA	Oligo probe to detect Eco *RydC*
KFO-0796	CTACGGCGTTTCACTTCTGAGTTC	Oligo probe to detect Eco *5S rRNA*
KFO-0797	GTTGATGGGCTCCACAA	Oligo probe to detect Eco *RybB*
KFO-0798	GTTCTGTACAACAACCTGGG	construction of plasmid pKF491-8
KFO-0799	CTGCAGGAAGGGGATCCG	construction of plasmid pKF491-8
KFO-0826	CCGTATGTAGCATCACCTTC	Oligo probe to detect *gfp*
KFO-0852	GTACAATTGAGACGTATCG	T7 template *hfq* riboprobe
KFO-0853	GTTTTTTTTAATACGACTCACTATAGGGAGG TCT CTC TTT TCC TTA TAT GC	T7 template *hfq* riboprobe

Table S4. All strains used in this study.

Strain	Stock name	Bacterium	Genotype/relevant markers	Reference
***Ec* Hfq**	KFS-0191	*E. coli*	MC4100 wild-type	Silhavy laboratory, Princeton University
***Ec*65 Hfq**	KFS-0705	*E. coli*	MC4100 P*hfq::Ec_hfq65::FRT*	this study
***Cc* Hfq**	KFS-0706	*E. coli*	MC4100 P*hfq::Cc_hfq::FRT*	this study
**Δ*hfq***	KFS-0708	*E. coli*	MC4100 Δ*hfq::FRT*	this study
**TOP10**		*E. coli*	TOP10	Invitrogen
**ER2566**		*E. coli*	ER2566	NEB
**NA1000**	KFS-0006	*C. crescentus*	NA1000	Gitai laboratory, Princeton University
**BL21(DE3)**		*E. coli*	B F^{-} *ompT gal dcm lon hsdS*$_B$($r_B^{-}m_B^{-}$) λ(DE3 [*lacI lacUV5-T7p07 ind1 sam7 nin5*]) [*malB*$^+$]$_{K\text{-}12}$(λ^S)	Wood, 1966
**NM694**		*E. coli*	BL21(DE3) Δ*hfq::cat-sacB*	Santiago-Frangos et al, 2016
**AZ233**		*E. coli*	MC4100 miniλ Δ*hfq::cat-sacB* Δ*term*	Zhang et al, 2013

Table S5. All plasmids used in this study.

trivial name	plasmid ID	description	origin/marker	reference
	pXG10	Empty vector	pXG10/CamR	(Urban & Vogel, 2007)
	pTYB11	Empty vector	pBR322/AmpR	NEB
**pP$_{BAD}$*ctrl.***	pKP8-35	P$_{BAD}$ control plasmid	pBR322/AmpR	PMID 17427289
**pP$_{BAD}$-RydC**	pKF524-1	expresses *E. coli* RydC from arabinose-inducible P$_{BAD}$ promoter	pBR322/AmpR	this study
**pP$_{BAD}$-RydC**	pKF525-12	expresses *E. coli* DsrA from arabinose-inducible P$_{BAD}$ promoter	pBR322/AmpR	this study
**pP$_{BAD}$-RybB**	pKF529-6	expresses *E. coli* RybB from arabinose-inducible P$_{BAD}$ promoter	pBR322/AmpR	this study
**P$_{LtetO}$*ompF::gfp***	pSK05	expresses *E. coli ompF::gfp* translational fusion (-50 rel. to AUG + 13 codons of *ompF*) from constitutive P$_{LtetO-1}$ promoter	pXG10/CamR	PMID 17264113
**P$_{LtetO}$*hns::gfp***	pSK09	expresses *E. coli cfa::gfp* translational fusion (-36 rel. to AUG + 28 codons of *hns*) from constitutive P$_{LtetO-1}$ promoter	pXG10/CamR	PMID 17264113
**P$_{LtetO}$*cfa::gfp***	pKF206-1	expresses *E. coli cfa::gfp* translational fusion (-211 rel. to AUG + 15 codons of *cfa*) from constitutive P$_{LtetO-1}$ promoter	pXG10/CamR	(Fröhlich et al., 2013)
	pKF491-8	Expresses truncated *C. crescentus hfq* (aa 1-78) for purification with the IMPACT system	pBR322/AmpR	This study
	pKD4	Template plasmid for KmR mutant construction	oriRγ/AmpR, KanR	(Datsenko & Wanner, 2000)
	pKD46	Temperature-sensitive plasmid to express λRED-recombinase from arabinose-inducible P$_{araB}$ promoter	oriR101/ AmpR	(Datsenko & Wanner, 2000)
	pCP20	Temperature-sensitive Flp recombinase expression plasmid	pSC101/ AmpR, CamR	(Cherepanov & Wackernagel, 1995)
	pGEX-6p1-Cc Hfq	Expresses full length *C. crescentus* Hfq with a cleavable N-terminal GST tag.	AmpR	This study
	petDUET1-Cc Hfq	Expresses full length *C. crescentus* Hfq untagged	AmpR	This study
	petDUET1-EcCc Hfq	Expresses untagged chimera of *E. coli* Hfq core and *C. crescentus* Hfq CTD	AmpR	This study
	pET21b-Hfq	Expresses untagged wildtype *E. coli* Hfq	AmpR	Zhang et al, 2002
	pET21b-Hfq65	Expresses untagged *E. coli* Hfq core (N-terminal residues 1-65)	AmpR	Santiago-Frangos et al, 2016

Table S6. Titration series used for RNA gel shifts.

RNA	Hfq	Lane										
		1	2	3	4	5	6	7	8	9	10	11
R157		Hfq concentration (nM hexamer)										
	Ec	0.0	13.3	16.7	20.0	26.7	33.3	40.0	46.7	60.0	83.3	
	EcCc	0.0	13.3	26.7	40.0	53.3	60.0	83.3	150.0	250.0		
	Ec65	0.0	13.3	20.0	26.7	40.0	83.3	166.7	333.3	666.7	1333.3	
	Cc	0.0	13.3	20.0	26.7	33.3	40.0	46.7	53.3	60.0	83.3	
	Cc78	0.0	20.0	50.0	83.3	116.7	133.3	200.0	266.7	333.3	416.7	
R133												
	Ec	0.0	16.7	33.3	66.7	133.3	250.0	366.7	666.7	1333.3		
	EcCc	0.0	60.0	166.7	250.0	333.3	666.7					
	Ec65	0.0	13.3	20.0	26.7	40.0	83.3	166.7	333.3	666.7	1333.3	
	Cc	0.0	26.7	40.0	53.3	83.3	116.7	150.0	216.7	300.0	666.7	
	Cc78	0.0	83.3	166.7	266.7	333.3	416.7	500.0	666.7	833.3		
ChvR												
	Ec	0.0	33.3	66.7	133.3	200.0	250.0	366.7	666.7	1333.3		
	EcCc	0.0	60.0	166.7	250.0	333.3	666.7					
	Ec65	0.0	13.3	20.0	26.7	40.0	83.3	166.7	333.3	666.7	1333.3	
	Cc	0.0	666.7	1333.3								
	Cc78	0.0	83.3	166.7	266.7	333.3	416.7	500.0	666.7	833.3	1000.0	
RydC												
	Ec	0.0	13.3	20.0	26.7	33.3	40.0	46.7	53.3	60.0	83.3	
	EcCc	0.0	40.0	83.3	150.0	216.7	300.0	666.7	1333.3			
	Ec65	0.0	13.3	20.0	26.7	33.3	40.0	46.7	53.3	60.0	83.3	
	Cc	0.0	40.0	83.3	150.0	216.7	300.0	666.7	1333.3			
	Cc78	0.0	83.3	166.7	266.7	333.3	416.7	500.0	666.7	833.3	1000.0	
DsrA												
	Ec	0.0	13.3	20.0	26.7	33.3	40.0	46.7	53.3	60.0	83.3	
	EcCc	0.0	13.3	20.0	26.7	33.3	40.0	46.7	53.3	60.0	83.3	
	Ec65	0.0	13.3	20.0	26.7	33.3	40.0	46.7	53.3	60.0	83.3	
	Cc	0.0	13.3	20.0	26.7	33.3	40.0	46.7	53.3	60.0	83.3	
	Cc78	0.0	13.3	20.0	33.3	50.0	83.3	116.7	200.0	266.7	416.7	
ChiX												
	Ec	0.0	13.3	20.0	26.7	33.3	40.0	46.7	53.3	60.0	83.3	
	EcCc	0.0	13.3	20.0	26.7	33.3	40.0	46.7	53.3	60.0	83.3	
	Ec65	0.0	13.3	26.7	40.0	53.3	60.0	83.3	166.7	333.3	666.7	
	Cc	0.0	13.3	20.0	26.7	33.3	40.0	46.7	53.3	60.0	83.3	
	Cc78	0.0	13.3	20.0	33.3	50.0	83.3	116.7	200.0	266.7	416.7	
RybB												
	Ec	0.0	0.4	0.8	1.6	3.1	6.3	12.5	25.0	50.0	100.0	200.0
	EcCc	0.0	1.0	2.0	3.9	7.8	15.6	31.3	62.5	125.0	250.0	500.0
	Ec65	0.0	0.4	0.8	1.6	3.1	6.3	12.5	25.0	50.0	100.0	200.0
	Cc	0.0	0.6	1.2	2.3	4.7	9.4	18.8	37.5	75.0	150.0	300.0

	Cc78	0.0	0.8	1.6	3.1	6.3	12.5	25.0	50.0	100.0	200.0	400.0
cfa												
	Ec	0.0	0.4	0.8	1.6	3.1	6.3	12.5	25.0	50.0	100.0	200.0
	Cc	0.0	0.4	0.8	1.6	3.1	6.3	12.5	25.0	50.0	100.0	200.0
hns												
	Ec	0.0	0.4	0.8	1.6	3.1	6.3	12.5	25.0	50.0	100.0	200.0
	Cc	0.0	0.4	0.8	1.6	3.1	6.3	12.5	25.0	50.0	100.0	200.0

References

1. Zhang A, Wassarman KM, Ortega J, Steven AC, Storz G (2002) The Sm-like Hfq protein increases OxyS RNA interaction with target mRNAs. *Mol Cell*. **9**:11-22.

2. Santiago-Frangos A, Kavita K, Schu DJ, Gottesman S, Woodson SA (2016) C-terminal domain of the RNA chaperone Hfq drives sRNA competition and release of target RNA. *Proc Natl Acad Sci U S A.* **113**:E6096.

3. Kleiger G, Saha A, Lewis S, Kuhlman B, Deshaies RJ (2009) Rapid E2-E3 assembly and disassembly enable processive ubiquitylation of cullin-RING ubiquitin ligase substrates. *Cell* **139**:957-68

4. Santiago-Frangos A, Jeliazkov JR, Gray JJ, Woodson SA (2017) Acidic C-terminal domains autoregulate the RNA chaperone Hfq. *Elife* **6**:e27049

5. Tyka MD, Keedy DA, Andre I, Dimaio F, Song Y, et al. (2011) Alternate states of proteins revealed by detailed energy landscape mapping. *Journal of molecular biology* **405**: 607–618

6. Chaudhury S, Lyskov S, Gray JJ (2010) PyRosetta: a script-based interface for implementing molecular modeling algorithms using Rosetta. *Bioinformatics*, **26**: 689-691

7. Alford RF, Leaver-Fay A, Jeliazko JR, O'Meara MJ, DiMaio FP, Park H, Shapovalov MV, Renfrew PD, Mulligan VM, Kappel K, et al (2017) The Rosetta all-atom energy function for macromolecular modeling and design. *J Chem Theory Comput*. **13**:3031-3048

8. Mclachlan AD (1982) Rapid comparison of protein structures. *Acta Cryst.* **A38**:871-873

9. Sagawa S, Shin JE, Hussein R, Lim HN (2015) Paradoxical suppression of small RNA activity at high Hfq concentrations due to random-order binding. *Nucleic Acids Res*. **43**:8502-15

10. Rappsilber J, Ishihama Y, Mann M (2003) Stop and go extraction tips for matrix-assisted laser desorption/ionization, nanoelectrospray, and LC/MS sample pretreatment in proteomics. *Anal Chem.* **75**:663-70

11. Craig R, Beavis RC (2004) TANDEM: matching proteins with tandem mass spectra. *Bioinformatics* **20**:1466-7

12. Dorfer V, Pichler P, Stranzl T, Stadlmann J, Taus T, Winkler S, Mechtler K. MS Amanda, a universal identification algorithm optimized for high accuracy tandem mass spectra. *J Proteome Res*. **13**:3679-84

13. Kim S, Pevzner PA (2014) MS-GF+ makes progress towards a universal database search tool for proteomics. *Nat Commun*. **5**:5277

14. Vaudel M, Barsnes H, Berven FS, Sickmann A, Martens L (2011) SearchGUI: An open-source graphical user interface for simultaneous OMSSA and X!Tandem searches. *Proteomics* **11**:996-9

15. Vaudel M, Burkhart JM, Zahedi RP, Oveland E, Berven FS, Sickmann A, Martens L, Barsnes H (2015) PeptideShaker enables reanalysis of MS-derived proteomics data sets. *Nat Biotechnol*. **33**:22-4

16. Antal M, Bordeau V, Douchin V, Felden B. (2005) A Small Bacterial RNA Regulates a Putative ABC Transporter. *J. Biol. Chem*. **280**:7901-8

17. Panja S, Woodson SA. (2015) Fluorescence reporters for Hfq oligomerization and RNA annealing. *Methods Mol Biol*. **1259**: 369-83

18. Małecka EM, Stróżecka J, Sobańska D, Olejniczak M. (2015) Structure of bacterial regulatory RNAs determines their performance in competition for the chaperone protein Hfq. *Biochemistry*. **54**:1157-70
